# Private, non-profit, and plantation: Oil palm smallholders in management-assistance programs vary in socio-demographics, attitudes, and management practices

**DOI:** 10.1371/journal.pone.0304837

**Published:** 2025-01-17

**Authors:** Valentine J. Reiss-Woolever, Wakhid Wakhid, Muhammad Ikhsan, Jean-Pierre Caliman, Muhammad Naim, Elfina N. Azmi, Sharyn Shufiyan, John Howes, Reza Azmi, Ying Ying Lim, Siti Zulaikah Abdul Jan, Isaac Barrock, Badrul Azhar, Julia Drewer, Caroline Ward, Joshua A. Jones, Sarah H. Luke, Edgar C. Turner, Purnama Hidayat, Damayanti Buchori

**Affiliations:** 1 Department of Zoology, Insect Ecology Group, University of Cambridge, Cambridge, United Kingdom; 2 Department of Agrotechnology, Faculty of Agriculture, Tribhuwana Tunggadewi University, Malang, Indonesia; 3 Department of Plant Protection, IPB University, Bogor, Indonesia; 4 Sinar Mas Agro Resources Technology Research Institute (SMARTRI), Pekanbaru, Riau, Indonesia; 5 Wild Asia, Kuala Lumpur, Malaysia; 6 Department of Forest Science and Biodiversity, Faculty of Forestry and Environment, Universiti Putra Malaysia, Selangor, Malaysia; 7 UK Centre for Ecology & Hydrology, Penicuik, United Kingdom; 8 Leverhulme Centre for Anthropocene Biodiversity, University of York, York, United Kingdom; 9 Department of Biology (Mansfield Road), University of Oxford, Oxford, United Kingdom; 10 School of Biosciences, University of Nottingham, Sutton Bonington Campus, Nr Loughborough, United Kingdom; 11 Center for Transdisciplinary and Sustainability Sciences, IPB University, Bogor, Indonesia; Federal University of Agriculture Abeokuta, NIGERIA

## Abstract

Smallholder farmers produce over 40% of global palm oil, the world’s most traded and controversial vegetable oil. Awareness of the effects of palm oil production on ecosystems and human communities has increased drastically in recent years, with ever louder calls for the private and public sector to develop programs to support sustainable cultivation by smallholder farmers. To effectively influence smallholder practices and ensure positive social outcomes, such schemes must consider the variety in perspectives of farmers and align with their priorities. We conducted social surveys on smallholder farmers in Indonesia and Malaysia with varying degrees of participation in programs that offer advice and support with plantation management (“management-assistance programs”) led by an industrial palm oil producer in Indonesia and a conservation-focused NGO in Malaysia. We surveyed farmers on their demographics, attitudes, and management decisions. Our analyses act as case studies to investigate the similarities and differences between smallholder palm oil producers involved in different schemes, allowing us to determine the alignment between the intentions of partnership programs and the current realities of smallholder plantations. The relationship between heterogeneity of social factors and management decisions and degree of program involvement differed across different groups and region: Indonesian smallholders most closely partnered with the private sector were the most varied in socio-demographics and attitudes but showed little variation in management inputs, while Malaysian smallholders most closely partnered with an NGO were the most heterogenous across all survey sections. Specifically, Indonesian farmers partnered with the private sector used less herbicide, more fertilizer, and had higher yield and total household income than farmers completely uninvolved with management assistance programs. In Malaysia, farmers partnered with an NGO also had higher yield and fertilizer application than independent farmers, however they used significantly more herbicide and had lower total household income. Our findings demonstrate the wide variety of smallholder farmers in both regions, directly opposing a ‘one-size-fits-all’ approach to sustainability. The wide variety of existing management practices also provides a potentially valuable natural experiment to identify high-yield, environmentally-friendly management approaches. When taken in context, our findings may inform the interventions of management-assistance programs, ensuring they are approaching the most relevant farmer groups in the most effective way.

## Introduction

Palm oil (the processed product of oil palm, *Elaeis guineenis*) is the world’s cheapest and most widely used vegetable oil. Oil palm is cultivated on over 19 million hectares of land across the tropics [1], with current global production 35 times higher than in the 1970s [2]. While the industry contributes to local and national economies, there are significant concerns about its negative effects on biodiversity and ecosystem functioning, as well as on human communities and livelihoods [3, 4].

Over 85% of global palm oil production takes place in Malaysia and Indonesia, where smallholder farmers are responsible for an estimated 40% of production [5]. Smallholdings are farms smaller than 50 hectares, however they are typically two-to-three hectares in size in Malaysia and Indonesia, and are often backyard enterprises operated by household members simultaneously employed in other industries [6]. The global palm oil industry has been linked to severe negative impacts on biodiversity [7], climate change [8], and the land rights of local people [9], however it has also benefited farming communities in other contexts through employment and development [3]. It is widely agreed that smallholder cultivation is an essential part of the global supply of palm oil, although one which often operates below optimal production. The yield gap between smallholder and industrial plantations has been estimated at 50% [10], resulting in some instances in poor livelihood outcomes for smallholders, and increased need for land transformation to meet global palm oil demand. These yield gaps between individual plantations, regions, and industrial and smallholder plantations can be caused by unavoidable factors such as climate conditions and water availability [11]. However, they are often due to a slack of information and resources by smallholders to facilitate best practice, such as access to high quality seeds, adequate fertiliser and information on the most effective disease and pest prevention techniques [11].

As awareness of the ecological and social costs of oil palm has spread to consumers, there has been an increase in demand for more sustainable and traceable oil palm cultivation, leading to the development of sustainability certification schemes [12]. The Roundtable on Sustainable Palm Oil (RSPO), Indonesia Sustainable Palm Oil (ISPO), and Malaysian Sustainable Palm Oil (MSPO) systems all provide social and environmental guidelines aiming to facilitate efficient and sustainable production by both smallholder and industrial plantations [13]. These guidelines, which include principles related to social, environmental, and supply chain transparency, must be followed to receive certification. Such sustainability schemes aim to reduce the negative impacts of palm oil production by increasing efficiency along all stages of production, decreasing harmful impacts of plantation inputs on the environment, and improving the living conditions and livelihoods of farmers [14]. However, there is little information on their success, with only 15 publications on the social and/or ecological effects of oil palm certification schemes [15].

At a cultivation level, certification schemes aim to improve best practice, supporting plantations with information and resources to preserve remaining in-plantation biodiversity and increase yield, therefore decreasing the need for expansion into forested areas [16]. Unfortunately, smallholders often fall short of certification requirements due to a lack sufficient resources and knowledge, and are therefore at risk of being excluded from the sustainable palm oil market [13]. To combat this, numerous initiatives have been implemented by private, public, and non-governmental organisations to increase smallholder compliance capacity [17]. Smallholders involved in such initiatives can vary in the degree and direction of their partnership, from receiving land and broad information on management from government programs (such as FELDA, The Federal Land Development Authority, in Malaysia), to receiving direct application of inputs on a weekly basis from industrial plantations including by large-scale companies (such as Wilmar International or Sime Darby), or being trained in sustainability guidelines by NGOs (such as PanEco). There is also limited information on these “plasma” programs, with only 5% of ecological studies in oil palm involving such farmers [15].

On the other end of the spectrum are fully independent smallholders, who are not involved with any organization or government scheme and are autonomous in their plantation management strategies and decisions [18]. This may be due to personal choice, recent involvement in the industry, or the absence of management-assistance programs in their area. For independent farmers, plantation inputs may be decided on a day-by-day basis in response to changes in personal and social circumstances, rather than via consistent programs implemented over large areas [19]. Their decisions are influenced by a range of conditions including social pressures, socio-demographic and economic factors, cultural values, connection to nature, and personal history, and are often poorly understood by other industry stakeholders [20–22]. Independent smallholders must constantly conduct cost-benefit analyses as they manage the trade-offs between farm inputs, long-term investments, environmental sustainability, and household economic security [23]. As autonomous actors, independent farmers directly influence their plantations through their management decisions, and are directly influenced by their plantations through palm oil sales, sustenance crop yield, human health effects of inputs, and time dedicated to labour [24]. While the effect of implementation of sustainability schemes on crop productivity and ecosystem health has been investigated in previous papers [25–27], little research has been done to understand the socio-economic, personal, and plantation-level variation between farmers involved in management-assistance programs [15].

In this study, we aim to determine whether trends and relationships between demographics, attitudes, and management decisions are aligned with farmer involvement in management-assistance programs. This paper does not quantify the effects of such partnerships. Instead, we use a case study approach to determine the differences and similarities between smallholder palm oil producers involved in different schemes, to determine the alignment between the intentions of assistance programs and the current realities of smallholder plantations. To do so, we conducted mixed-method surveys with 95 individual smallholder farmers in Indonesia and Malaysia with varying degrees of involvement with management-assistance programs (an industrial palm oil producer and a conservation focused NGO, respectively) as examples of the patterns in, and heterogeneity of, farmer demographics, attitudes, and management decisions for these specific contexts. Similar surveys have been used to understand the relationship between farmer socio-demographics and agricultural inputs [28], socio-cultural motivations and forest management [29], risk attitude and participation in conservation agriculture [30], as well as experience, attitudes and crop choice [31]. This project involves an international team with expertise in the oil palm industry, including academic institutions (e.g., Institut Pertanian Bogor, henceforth referred to as IPB, partners in Indonesian surveys), NGOs (Wild Asia, a non-profit social enterprise, partners in Malaysian surveys), and the private sector (Sinar Mas Agro Resources and Technology Research Institute, PT. SMART R&D division, henceforth referred to as SMARTRI, partners in Indonesian surveys). We address the following key questions:

- What are the relationships and trends between smallholder socio-demographics, attitudes and opinions, and plantation management inputs?
- How do any emerging groupings and trends relate to degree of involvement of smallholders in partnership schemes?

## Methods

### Summary

We conducted surveys among 46 smallholders in Indonesia and 49 smallholders in Malaysia with three increasing degrees of external partnership involvement. The surveys included questions on the farmers’ socio-demographic characteristics, livelihoods, opinions and priorities towards nature and the oil palm industry, decision making factors, plantation management inputs, and yield.

The participants were selected on the basis of their being in an existing relationship with our local partner organizations (SMARTRI in Indonesia, Wild Asia in Malaysia) or not, location and ease of access, and equal distribution across the three categories of involvement in both regions. The location of participants and ease of access became particularly important due to government COVID mandates restricting travel, further limiting our potential pool and spread of participants. Because of this limitation, the farmer typologies are not ideally mixed spatially, and we were unable to specifically pair smallholders across the different strategies in space. While there were no other preferences for inclusion, within these constraints there was no additional choice of participants. The farmers were operating under their particular involvement category before our research began.

### Indonesian study sites

Indonesian surveys were conducted in the Kampar and Siak regencies of the Riau Province, on the island of Sumatra, Indonesia. Riau’s population is majority Melayu, with prominent Javanese, Minangkabau, and Chinese populations. Islam is the predominant religion amongst the 5.5 million inhabitants. Kampar has an area of 11,000 km^2^, and a population of approximately 840,000 [32]. Siak has an area of 8,500 km^2^ and a population of roughly 450,000 [32]. For both regions, the majority of the population works in agriculture, and palm oil production is one of the largest industries, with rapid expansion beginning in the 1980s.

Surveys in Indonesia were conducted by researchers from IPB University and SMARTRI, which is the research and development arm of Golden Agri Resources (GAR) through its plantation management company PT. SMART TBK, a large-scale industrial palm oil producer. SMARTRI currently advises 72,000 smallholder farmers over approximately 112,000 hectares of land (21% of their total processed land area). All SMARTRI related sites are currently certified sustainable by the RSPO.

### Indonesia study site farmer typology

**Fig 1:**
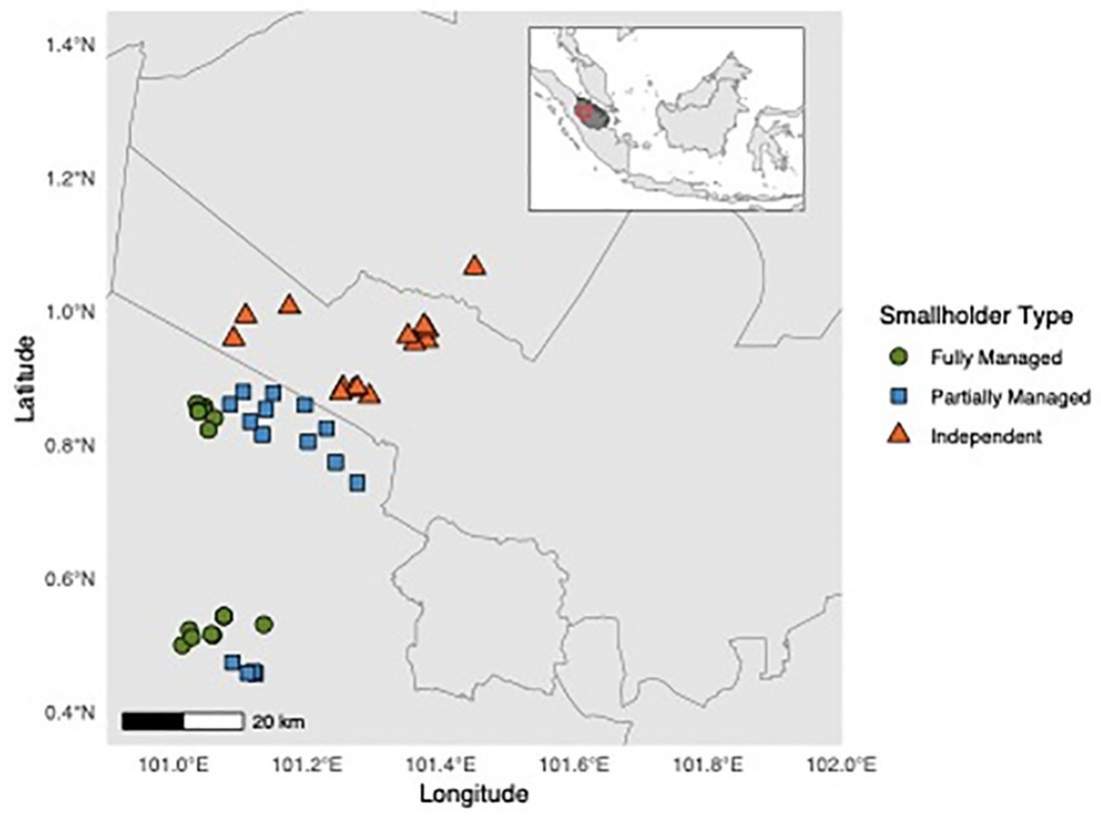
Map showing the distribution of Indonesian survey participant households by farmer typology, with insert showing larger geographical context. Green circles show "Fully-Managed” plantations, blue squares show “Partially Managed” plantations, and orange triangles show “Independent” plantations.

Within the Indonesian sites, the 46 farmers (Fig 1) were near-evenly distributed between three increasing levels of collaboration with SMARTRI:

Fully Managed farms (n = 16):
Survey participants who fall under the ‘Fully Managed’ categorization have the most involvement with industry in terms of extension services. The types, levels, and frequency of management inputs are decided upon and applied by SMARTRI, and farmers may not visit their plantations more than a few times a year. These farmers are akin to the ‘Nucleus Estate Smallholder’ (*Perkebunan Inti Rakyat)*, schemes implemented by Indonesian authorities in the 1970s with support from the World Bank [17], and are commonly known as ‘Plasma farmers’ within SMARTRI.
Partially Managed farms (n = 15):
Survey participants under ‘Partially Managed’ classification receive agricultural recommendations from SMARTRI, but the farmers must implement the inputs themselves, and are themselves responsible for cultivating their oil palm. Information given to smallholders in this category includes guidance on best practices for fertiliser management (e.g., type, doses levels, frequency), crop protection management including weed control (e.g., method, type, and frequency of herbicide application), and advice on harvesting techniques. Farmers may also receive information from other sources and are not on a set-management regime determined by SMARTRI as an outside party.
Independent farmers (n = 15):
Survey participants under ‘Independent’ classification do not receive any direct information or guidance from SMARTRI and have no relation to them as an industrial partner. They do not interact with SMARTRI regularly aside from the interviews we conducted. These farmers must decide upon, buy, and implement all inputs themselves, and are free to receive information from various sources (e.g., public sector information campaigns, farmer cooperatives or associations, private input through manufacturer advertising campaigns). These farmers are independent, and fully responsible for their own oil palm plantations.

### Malaysian study sites

Malaysian surveys were conducted in the Kampar District, Perak, in Peninsular Malaysia. The population of the Kampar District is approximately 100,000, with a majority Malay population and prominent Chinese and Indian minorities. The Kampar District is an area of approximately 670 km^2^. The district has a history of industrial production of natural reserves such as tin, and the economy is now largely commercial and industry focused. The study sites were based around Perak Tengah (Middle Perak) and Batang Padang, where palm oil production is the predominant industry.

Surveys in Malaysia were conducted by collaborators at Wild Asia who have been working with oil palm smallholders since 2012, and currently work with 1,294 smallholder producers. The farmers were operating under their particular involvement category before the study began.

### Malaysian study site farmer typology

**Fig 2:**
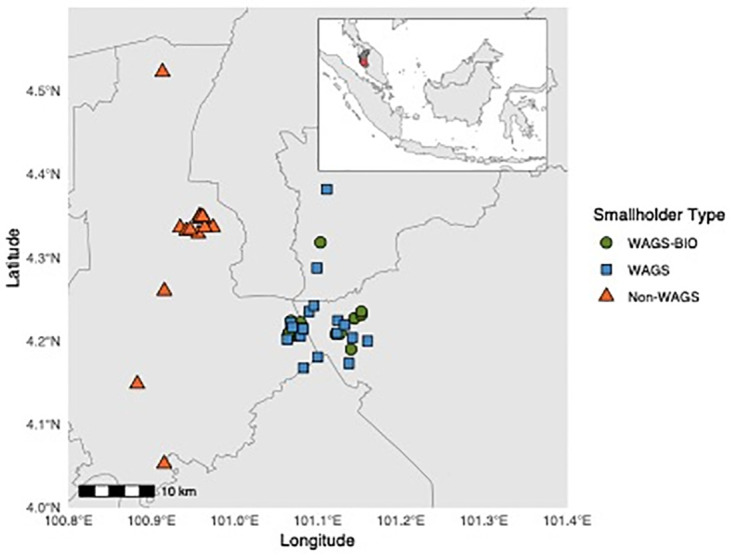
Map showing the distribution of Malaysian survey participant households by farmer typology, with insert showing larger geographical context.

Within the Malaysian sites, the 49 farmers (Fig 2) were near-evenly distributed between three increasing levels of collaboration with Wild Asia:

WAGS-BIO (n = 15):
Survey participants under the ‘WAGS-BIO’ (Wild Asia Group Scheme-BIO) classification are at different stages in the process of moving to a standard cultivation protocol developed by Wild Asia (from <1 to 5 years of participation). The protocol includes management methods which aim to reduce chemical and inorganic inputs, and foster more organic methods of cultivation, including use of cover crops. Farmers must demonstrate that their farms are not recent conversions, are not located on peat soils, and have three years of production and farm input records before being accepted into the WAGS-BIO program. Farmers participate in training workshops and have monthly visits from the Wild Asia farm team. Farmers also receive all of the information and training given to standard WAGS farmers (see below) and must have received RSPO and MSPO certification under the WAGS scheme before they are able to advance to WAGS-BIO. Of the three groupings, farmers in this category are most closely tied to Wild Asia and make few management decisions independently. However, they are responsible for applying management inputs themselves.
WAGS (n = 19):
Survey participants under the WAGS (Wild Asia Group Scheme) classification receive information on RSPO and MSPO certification requirements, with eventual RSPO technical assessments carried out by Wild Asia. Through their affiliation with Wild Asia, WAGS farmers have direct access to the global palm oil market. The farmers are given information on RSPO and MSPO best practice and training, however they must decide upon and implement the respective management inputs themselves.
Independent farmers (n = 15):
Survey participants under ‘Independent’ classification do not receive any information or guidance from Wild Asia and have no relation to them as an outside partner. They do not interact with Wild Asia regularly aside from the interviews we conducted. These farmers must decide upon, buy, and implement all management inputs themselves, and are free to receive information from various sources (e.g., government information campaigns, farmer cooperatives, input manufacturer advertising campaigns). These farmers are independent, and fully responsible for their own oil palm plantations. They are furthest in relation to Wild Asia of the three categories.

### Survey design

A semi-structured questionnaire was administered in the local language (Indonesian or Malay) between October and December 2021. The same, centrally-designed, survey was carried out in Indonesia and Malaysia. Interviews were done face-to-face where possible, and via phone to confirm and add missing information. Due to government COVID-19 restrictions on movement, seven interviews were conducted entirely over the phone. The questionnaire was designed after an initial scoping exercise in other sites in Malaysia (Banting, Malaysia; March, 2020), and consultation with the whole project team. The final questionnaire was approved by the University of Cambridge Department of Psychology Ethics committee in November 2021 (Application number ‘PRE.2020.004’).

Interviewers were trained in the study protocol including objectives of the study, rationale for specific questions, and interview techniques. Interviewers were from the study country and had previous experience in carrying out surveys of oil palm smallholders. The survey was structured into three sections (hereafter referred to as “survey sections”) with open- and close-ended questions. Close-ended questions included yes/no answers, ranking of options along a Likert scale, discrete quantitative value answers, and non-mutually exclusive multiple-choice questions. Long answer open-ended questions were not included in analyses but are sometimes quoted in the discussion to aid interpretation of quantitative findings. In the first section, we asked 21 questions regarding personal information and socio-demographic factors (hereafter referred to as ‘Socio-Demographic factors’). In the second section we asked 13 questions on attitudes towards nature, opinions on the oil palm industry, and decision-making factors and influences (hereafter referred to as ‘Attitudinal factors’). In the third section we asked 33 questions about plantation management, inputs, and yield (hereafter referred to as ‘Management Input factors’) (Table 1). While wage labourers are critical to oil palm cultivation in Southeast Asia, we surveyed only landowners and/or managers. As we aimed to investigate the factors which effected decision making, we spoke with the decision makers themselves, even when they may not have been those directly applying inputs. All results are self-reported by the farmers, and therefore have reporting inaccuracies and potential biases associated with them (see ‘Caveats to Study’ in Discussion section).

**Table 1:** Factors considered as part of the questionnaire and the rationale for their inclusion. Some factors were the focus of multiple questions in the final survey.

Factor	Definition	Rationale
Socio-demographic Factors		
Age	Age of the respondent	Farmer age plays a role in the level of previous farming experience, family obligation, manual labour capacity, openness to new technologies, and risk-taking behaviour, all of which may influence management decisions [33, 34].
Gender	Gender of the respondent (male or female)	Gender has links to land access, land ownership, and scale of production both in oil palm [35] and other smallholder crops [36]. The oil palm industry is typically dominated by men in Indonesia, mirroring employment levels in other crops and employment overall [19].
Education	The respondent’s furthest completed level of formal education (no-schooling to postgraduate)	Education level can affect plantations through its relationship with farmer openness to technology and new agricultural techniques, as well as pro-environmental behaviour [37, 38].
Household size	Number of adults and children in the household	A higher number of individuals per household may translate into greater labour availability for farm production activities. Previous studies on oil palm systems have found that smallholder farmers involved in certification programs generally have a significantly larger household number than uncertified farmers [39].
Oil palm income	Monetary value of the family’s income earned from oil palm cultivation	Smallholder farmers can be impoverished members of rural communities, with average monthly incomes of oil palm smallholders in Malaysia only RM 700 a month (122 GPB) [40]. High income oil palm smallholders have been reported to apply significantly higher inputs of fertiliser and labour in some studies [41], but with other studies reporting no relationship between income level and inputs [17].
Non-oil palm income	Monetary value of the family’s income earned from sources other than oil palm	Smallholder farmers often diversify their income with off-farm activities, to protect from changes in commodity markets and uncertainties from climatic conditions [40]. Previous studies have reported significant differences in plantation practices between farmers with higher non-agricultural incomes [41].
Landowner status	If the respondent owns the land, or it is owned by a larger organization	Famers who own their own land can spend more on external labour, and travel further for mill processing [35]. Ownership may determine a farmer’s likelihood to make long-term decisions, when considering inputs and environmental repercussions of cultivation.
History on land	Years that the respondent has personally used the land for farming	Previous studies have reported correlations between farmer age, education level, and crop preference with farmer residential history [33]. More recent movers to the location may have fewer kinship ties and less experience in similar landscapes, which may affect their interest and involvement in support schemes.
Attitudinal Factors		
Attitudes toward nature	How the respondent views nature, its benefits, and how they interact with wildlife on the plantation	Farmers with a more positive attitude and sense of connectedness to nature often make more environmentally conscious management decisions and often have a greater tendency to comply with certification sustainability standards [42].
Decision making factors	In their own opinion, what drives a farmer’s decisions on how to manage their plantation	Individuals make decisions based on personal history and experience, perceived societal expectations, and the level of information they receive from respected sources [43, 44]. Depending on what most heavily influences a farmer’s decision making process, their implementation of plantation inputs and cultivation techniques may favour different objectives.
Management Input Factors		
Planting density	The number of oil palms per area on the plantation, according to the respondent	Palm planting density influences the mortality rate and kilogram yield per palm, and therefore the environmental and economic sustainability of the plantation [33]. While oil palms are long-lived and replanted every 20–30 years, replanting frequency, planting density, and thinning practice are significant decisions made by smallholders which can influence plantations for decades.
Labour contribution	How many hours does the respondent, and their family members, spend on oil palm cultivation per week	Increased ease of cultivation, and therefore fewer hours worked in the plantation, has been identified as a reason smallholder farmers choose oil palm over other crops [45]. Limited labour capacity has been identified as a significant constraint to the intensity of production for smallholder farmers [46].
Soil additives	Does the respondent add any organic or inorganic additives to their soil	Soil quality is directly influenced by soil management decisions such as addition of empty fruit bunches and leaf litter. The resulting soil quality influences individual palm health, yield, and wider plantation environmental conditions [47].
Herbicide application	At which quantity, frequency, and method does the respondent apply herbicides	Herbicides are applied in oil palm estates to remove non-crop understory vegetation which may be competing with oil palms for resources or make harvesting more difficult. The herbicide type, method, and frequency of application is determined by individual farmers. These decisions have widespread effects, as herbicide application can increase yield, but also have secondary effects on human health and plantation biodiversity [48, 49].
Clearing practice	Does the respondent clear vegetation in the plantation- if yes, using what method and at what frequency	Vegetation clearing is done to reduce understory plant cover, and manually eliminate weeds which complete for resources with crop species. The degree of clearing practice undertaken by each farmer has effects on yield, plant and animal biodiversity, soil quality, and decomposition [50–53].
Livestock	Are there livestock present on the plantation- if yes, what types	Incorporating livestock into oil palm plantations has the potential to increase economic stability and environmental sustainability of oil palm plantations [54]. However, it may involve more intricate socio-economic and environmental factors than crop-only plantations. The decision to maintain livestock, and calculate the trade-offs involved, is a significant decision made by each smallholder.
Fertiliser application	What type of fertiliser does the farmer use, and with what frequency do they apply it	Effective fertiliser application can increase oil palm productivity and support the ecological sustainability of a plantations [46]. However, when implemented ineffectively it can be financially costly and damage the surrounding environment [47, 55]. The fertiliser type, application frequency, and volume are decided upon by each individual farmer and can vary greatly.
Intercropping	Is the plantation only oil palm, or does the smallholder cultivate other crops in the same area–if yes, which crops and why	Intercropping involves planting non-oil palm crop species in the same cultivation area. This practice is more common in smallholder than industrial systems [54], and can provide a more economically robust plantation, diverse sustenance opportunities, and ecological benefits.
Yield	What is the monthly yield per hectare in kilograms from the plantation	One of the most pressing issues in oil palm cultivation is the yield gap between smallholder and industrial estates [33]. This reduced productivity has income effects for the farmers, as well as wider environmental impacts, as more land is needed to meet global palm oil demand [16]. More targeted management, made through better informed management decisions, may help achieve best practice, and increase smallholder productivity [11, 17].

While the surveys were designed centrally, they were carried out locally by different regional research teams. Teams were unable to co-practice conducting surveys due to restricted travel between countries, owing to COVID-19 government restrictions. Surveys were therefore conducted in different contexts and occasionally varied in which questions were confidently answered and which were omitted, and thus we have not carried out any specific analyses between the two locations, but instead discuss results together in the discussion. Surveys were translated into English by field teams and checked within the research team.

### Data processing

Malaysian farmers under WAGS-BIO classification varied in the duration of their involvement with the WAGS-BIO program (from 5 to <1 years). Sensitivity analysis showed that the duration of involvement in WAGS-BIO did not have a statistically significant effect on any survey section, and therefore farmers were treated as one group.

Village and district identities were used to categorise geographic location rather than distances from a given site, due to the small spread in site distances, and the strong influence of village and district communities on farming practices reported across smallholders [24, 56]. To facilitate comparisons across farms of different sizes, all quantitative management inputs (such as herbicide amounts or labour hours) were adjusted to units per hectare. For missing responses on quantitative management inputs, due to lack of respondent knowledge or engagement, averages for the relevant farmer typology were used (Indonesian study sites n = four missing responses, Malaysian study sites n = two missing responses). Data management was conducted identically for Indonesian and Malaysian sites across all farmer typologies. A clean and anonymised version of the data is available via the Environmental Information Data Centre (S1 Text).

### Statistical analyses

All statistical analyses were performed in R version 4.0.2 within R Studio version 1.4.456 [57]. We used tidyverse [58], dplyr [59], and reshape2 [60] for data wrangling and visualization. Unless otherwise stated, all figures were plotted using ggplot2 [61].

To determine the potential effect of spatial correlation on responses, we tested for distance-decay of similarity using a permutational Mantel test (R package “vegan” [62]). All R values for socio-demographic, attitudinal, and management input variables were within ±0.07 of 0, therefore showing no strong positive nor negative spatial correlation. We therefore did not include any specific spatial information in later analyses [63].

As all quantitative values (e.g., yield, herbicide and fertiliser volumes) were self-reported by farmers, there is potential for uncertainty and unintentional inaccurate data. We therefore used ordinations to reduce the impact of inaccurately reported individual responses. To determine the similarities and differences in socio-demographic, attitudinal, and management decision factors between sites, we used Principal Component Analysis (PCA) [63] independently, for each of the three survey sections on scaled data. All sites were included (Indonesia: n = 46 sites, Malaysia: n = 49 sites) and coded to one of the three typographies separately for each country. This resulted in three separate PCA plots for each region, with ANOVA analyses run to determine the contribution of each factor to each ordination. We tested for significant differences between groups using ANOSIM with a Bray-Curtis dissimilarity matrix with 999 permutations, using ‘vegan’.

We linked the socio-demographic, attitude, and plantation input survey sections by conducting Canonical Correspondence Analyses (CCA), to quantify the relationships between the factors of two survey sections at a time. This included the following CCA models: socio-demographic factors as the independent variables, and attitudinal factors as the dependent variables; socio-demographic factors as the independent variables, and management decision factors as the dependent variables; attitudinal factors as the independent variables, and management decision factors as the dependent variables. To account for potential multicollinearity, the highest Variance Inflation Factors were eliminated until all had a VIF under ten. Once again, ANOVA tests were run to determine which factors had the largest impact on CCA output.

## Results

### Indonesian study sites

#### Summary of results

The extent of heterogeneity within and between farmer typologies varied between survey sections. There was large spread in socio-demographic responses within the typologies, particularly in Fully Managed farms, and less variation in management inputs within Fully and Partially Managed farmers than Independent farmers. Socio-demographics affected management inputs, in particular geographic and socio-economic factors.

#### Average sociodemographics of Indonesian farmers

**Fig 3:**
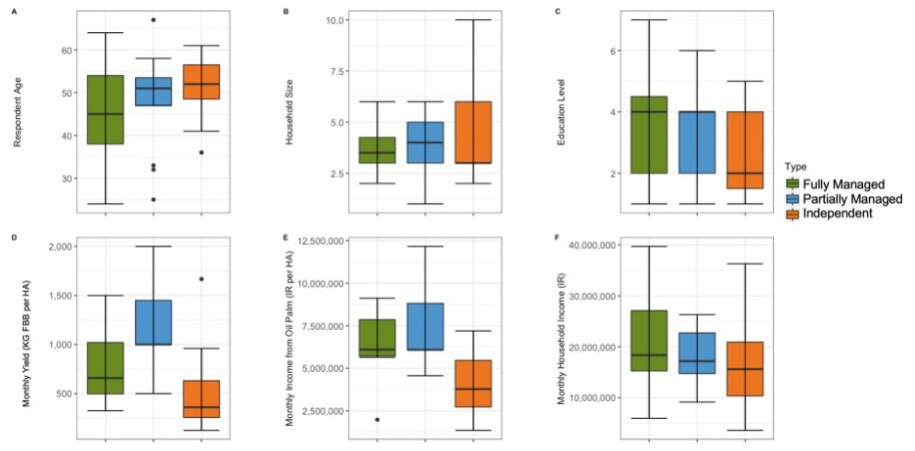
Comparisons of farmer responses in Indonesia for socio-demographic questions with numerical responses (A-F). Median, interquartile range, range, and outliers are shown.

The average and variation in age of respondents was similar for Partially Managed (Average = 48.4, SE = 2.8) and Independent farmers (Average = 51.7, SE = 1.8), with a lower average (Average = 45.4, SE = 2.7) and greater variation in farmer age seen in Fully Managed farmers (Fig 3A). The average household size was similar for the three typologies (Fully Managed: Average = 3.7, SE = 0.28; Partially Managed: Average = 3.9, SE = 0.38; Independent: Average = 3.6, SE = 0.61), with the largest household in an Independent farm (10), the smallest in a Partially Managed farm (1), and most variation in Independent farms (Fig 3B). The average education level was similar across groups, with the lowest level of education constant for all three farmer typologies (Elementary School), and the highest level of education seen in Fully Managed farmers, with a Masters Degree (Fig 3C). Average monthly yield in kilograms per hectare varied between typologies, and was highest in Partially Managed farms (Average = 1171.6, SE = 107.2), followed by Fully Managed farms (Average = 762.3, SE = 84.3), and was lowest in Independent farms (Average = 520.4.7.3, SE = 104.7). Partially Managed farms had the most variation in monthly yield per hectare. The highest yield per hectare was reported by a Partially Managed farmer (2000 KG per HA per month) (Fig 3D). The average monthly income from oil palm per hectare of land farmed was similar for managed farms (Fully Managed: Average = 6408775.5, SE = 411503.3; Partially Managed: Average = 7351500, SE = 586957.5) but lower in Independent farms (Average = 4118400, SE = 474551.5). The highest income from oil palm per hectare was reported by a Partially Managed farmer (12168000 IR) and was the highest outlier at over twice the average (Fig 3E). The average total monthly household income, from oil palm and other industries, was similar for the three Farmer Typologies (Fully Managed: Average = 20957762.5, SE = 2179643.6; Partially Managed: Average = 18214200, SE = 1332770.9; Independent: Average = 16024666.7, SE = 2179976), with a similar range for Fully Managed and Independent farmers, and the least variation in income within the Partially Managed group (Fig 3F).

#### Average management inputs of Indonesian farmers

**Fig 4:**
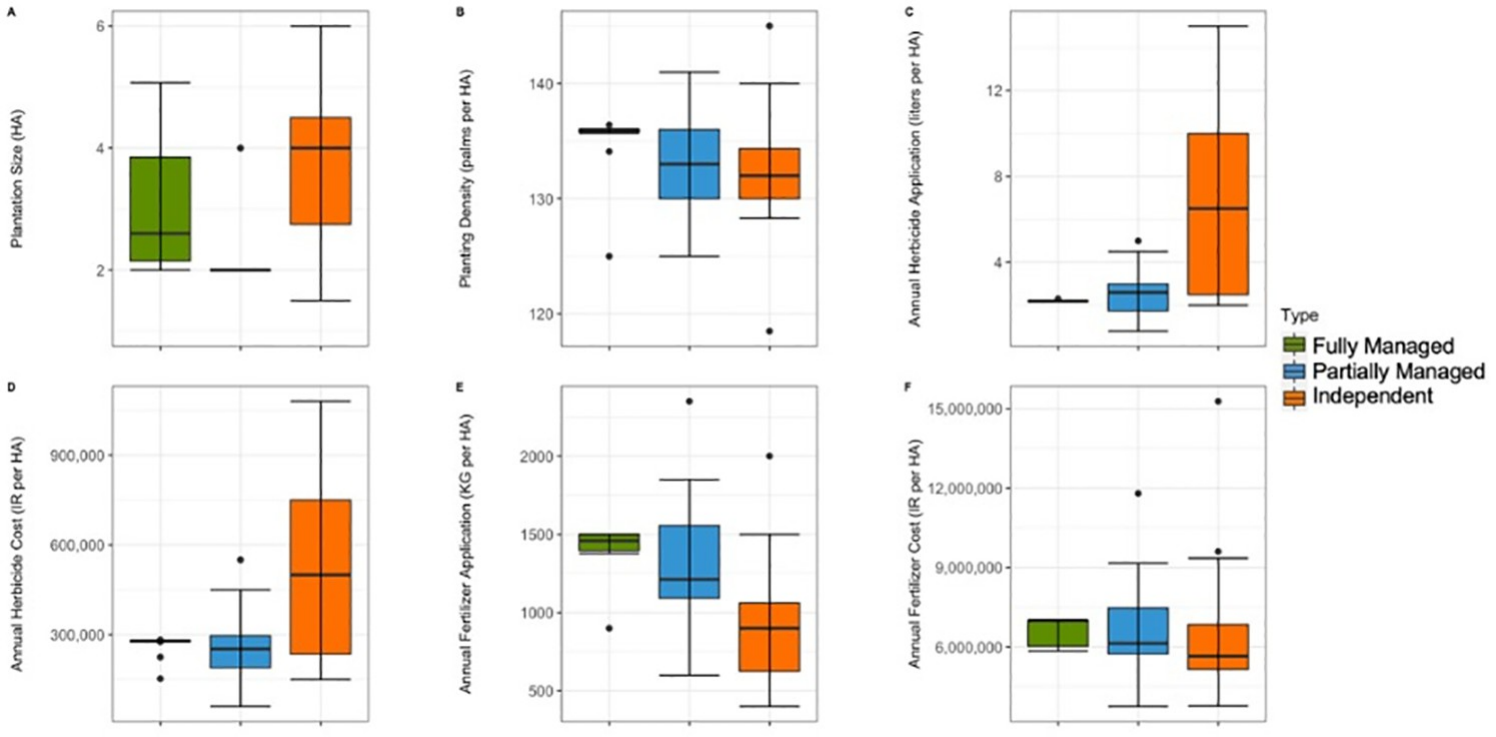
Comparisons of farmer responses for the Management Input questions with numerical responses (A-F) for Indonesian farmers. Median, interquartile range, range, and outliers are given.

The highest average plantation size was seen in Independent farms (Average = 3.6, SE = 0.4), which was nearly twice that of the other farmer typologies (Fully Managed: Average = 2.9, SE = 0.24; Partially Managed: Average = 2.1, SE = 0.1). The smallest plantations, and smallest range of plantations, was recorded in Partially Managed farms, of which all were 2 hectares, except one outlier of 4 hectares (Fig 4A). There was little variation in average palm planting density across the three farmer typologies (Fully Managed: Average = 135.2, SE = 0.7; Partially Managed: Average = 132.8, SE = 1.3; Independent: Average = 132.3, SE = 1.5), with 26 palms per hectare difference between the maximum (145 palms per hectare–Independent farmer), and minimum planting density (119 palms per hectare–Independent farmer). Fully Managed plantations had almost no variation in palm planting density (Fig 4B). While the average amount of herbicide applied per hectare was similar for the two Managed farmer groups (Fully Managed: Average = 2.2, SE = 0.02; Partially Managed: Average = 2.6, SE = 0.4), it was nearly three times higher for Independent farmers (Average = 6.5, SE = 1.1). There was little variation in herbicide application for Fully Managed farms, while the largest amount of variation was seen in Independent farms (Fig 4C). The average cost of herbicide per litre was also nearly twice as high in Independent farms than Managed farms (Fully Managed: Average = 268189.4, SE = 8406; Partially Managed: Average = 258807.9, SE = 42295.6; Independent: Average = 501538.5, SE = 80717.7), with nearly no variation in Fully Managed farms, some variation in Partially Managed farms, and the most variation in Independent farms (Fig 4D). Fully Managed farmers used the highest amount of fertiliser per hectare on average (Average = 1417.1, SE = 0.9), and Independent farmers used the lowest (Average = 942.1, SE = 0.8) (Fig 4E). Average fertiliser cost per hectare was similar for all three farmer typologies (Fully Managed: Average = 6591963.7, SE = 430650.8; Partially Managed: Average = 6568333.3, SE = 520194.7; Independent: Average = 6658611.1, SE = 745005.7), with the least amount of variation in Fully Managed plantations (Fig 4F).

#### Independent factors between site types

**Fig 5:**
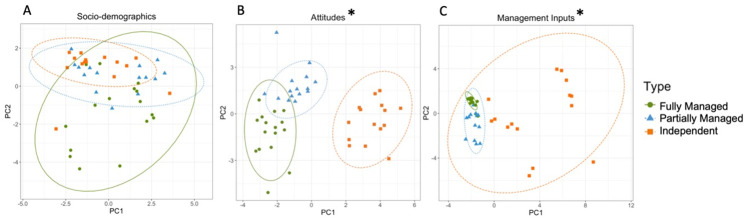
Results of principal component analyses, showing variation in questionnaire answers for socio-demographic (A) attitudinal (B), and management input (C) factors in Indonesia. Points are spherically grouped by farmer typology for the three different farmer typologies. The dotted line grouping each farmer typology is based on a 95% confidence interval. The Asterisk (*) represents statistically significant models (p > 0.05). Note: Scale differs between figures.

For socio-demographic factors, the first PCA component accounted for only 18% of variation, and the second component for 16%. There was substantial overlap between the three farmer typologies (Fig 5A), and there was no significant difference between farmer typologies (ANOSIM: R = 0.049, p = 0.06). Fully Managed farmers showed the highest variation in socio-demographic questions, with large variation across both PC1 and PC2. Partially Managed and Independent farmers however were skewed largely along PC1, which was positively correlated with farmer involvement in an industry other than agriculture and total household income, and negatively correlated with the proportion of total household income that was derived from agriculture. Fully Managed farmers split from other typographies in PC2, which was correlated with the proportion of total household income that was derived from agriculture, and respondent gender. There was a stronger correlation with respondents from PTP village (coded to maintain anonymity), than other villages (S1 Table).

For attitudes, preferences, and opinions, the first component accounted for 26% of variation, and the second component for 18%. There was a statistically significant difference between farmer typologies (ANOSIM R = 0.452, p = 0.0001). Independent farmers, the most heterogenous group, were distinct across PC1, while there was slight overlap between Fully and Partially Managed farmers on both axes (Fig 5B). The three farmer typologies were largely non-overlapping across the PC1 axis, which was positively correlated with a preference for fertiliser recommended by fertiliser suppliers over other influencing factors, such as the behaviour of neighbours or influence from cooperatives. It was also positively correlated with a cost-based decision-making process, and negatively correlated with a reported tendency to make decisions based on scientific advice. Partially Managed farmers were most homogenous across PC2, although all three farmer typologies displayed outliers across the PC2 axis which was most positively affected by a tendency to determine timing of herbicide treatment by observed presence of weeds, and a farmer’s preference for agriculture over other forms of employment. The second component was also negatively associated with a preference for fertiliser recommended by fertiliser suppliers over other influencing factors (S1 Table).

For management inputs, the first component accounted for 37% of variation, and the second for 14%. There was a statistically significant difference between farmer typologies (ANOSIM R = 0.1687, p = 0.0001), although Independent farmer responses fully encompassed both Fully and Partially Managed groups (Fig 5C). There were several magnitudes more variation across both axes in Independent farmers than the other two groups, while Fully Managed farmers showed very little variation in management input across either axis. Partially Managed farmers also showed limited variation across the PC1 axis, which was positively correlated with application of herbicide both along paths and around palms and higher amounts of herbicide applied, and negatively correlated with a greater number of oil palm harvests. Partially Managed and Independent farmers were both pulled along PC1, which meant they were associated with use of organic manure, non-chemical vegetation control methods (such as manual-cutting, which can be used alone or in conjunction with chemical vegetation control), and amount of herbicide used annually than Fully Managed farmers (S1 Table).

#### Relationships between factors by site type

**Fig 6:**
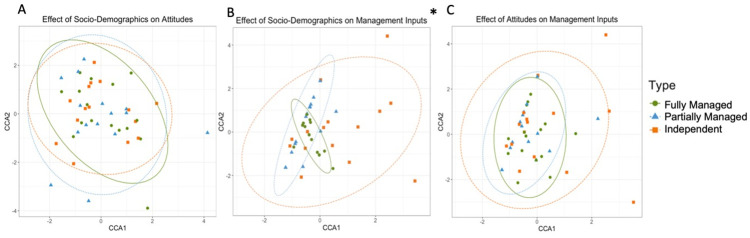
Results of Canonical Correspondence Analysis, showing the relationship between farmer responses on Socio-Demographics to Attitudes (A), Socio-Demographics to Management Inputs (B), and Attitudes to Management Inputs (C) in Indonesia. Points are spherically grouped by farmer typology for the three different farmer typologies. The dotted line grouping each Farmer Typology is based on a 95% confidence interval. The Asterisk (*) represents statistically significant models (p > 0.05). Note: Scale differs between figures.

Farmer socio-demographic factors did not significantly affect their attitudes (F = 1.02, p = 0.407). The first component explained only 18% of variation, while the second component explained 13% of variation. There was near complete overlap between the three groups, and all three Farmer Typologies displayed similar levels of heterogeneity across both axes (Fig 6A). The first component was significantly positively correlated with household income (F = 1.57, p = 0.048), and non-significantly positively correlated with participants’ village. It was most negatively correlated with the proportion of household income coming from agriculture, and larger household size. The second component was most positively affected by village, followed by household size and total household income plantation size, as well as household income. The attitudinal factors most affected by sociodemographic characteristics were the wildlife respondents felt most positively about seeing in their plantation (particularly leopard cats, wild pigs, long tailed macaques, weaver ants, and cobras). See S2 Table for highest factor loadings for dependent attitudinal factors, and S3 Table for descriptive statistics of the interaction between all questionnaire responses.

Socio-demographics had a significant effect on management inputs (F = 5.35, p = 0.002). The first component explained 82% of variation, while the second component explained only 2% of variation. Fully Managed and Partially Managed groups partially overlapped, and were both fully encompassed by the Independent farmer group (Fig 6B). The variation within Independent farms was markedly larger than that for Fully- or Partially- Managed farms, with Fully Managed farms displaying the most homogeneity. Independent farmers showed more variation along CCA1, which was significantly positively correlated with village LJ (F = 39.66, p = 0.001) and household size (F = 22.65, p = 0.001), and negatively correlated with monthly income from oil palm (F = 27.92, p = 0.001) and village KJ (F = 0.006, p = 0.033). Fully and Partially Managed farms displayed variation largely along the CCA2 axis, which was positively correlated with larger household size, and negatively correlated with village KJ. The management inputs most correlated to sociodemographic factors were organic manure use, non-herbicide vegetation control, plantation area, volume of herbicide used per hectare, and cost of herbicide per hectare. See S2 Table for highest factor loadings for dependent attitudinal factors, and S3 Table for descriptive statistics of the interaction between all questionnaire responses.

The effect of attitudinal factors on management inputs was not significant (F = 1.76, p = 0.16), and the first component explained 78% of variation, while the second component explained only 3% of variation. There was near complete overlap between groups, with ellipses for Fully Managed, Partially Managed, and Independent farmers forming concentric circles (Fig 6C). The greatest variation within farmer groups was seen in Independent farmers, with several outliers along both axes, and greater variation than other typologies along CCA1. The first component was positively correlated with negative attitudes to cobras in the plantation (F = 6.97, p = 0.022), rather than yield effecting wildlife. It was most negatively correlated with farmers who relied more heavily on scientific advice when making management decisions (F = 4.72, p = 0.050), as well as those who more highly valued the wildlife value of nature (F = 7.37, p = 0.030) and made primarily cost-based decisions (F = 6.92, p = 0.019). Fully and Partially Managed farmers showed similar levels of heterogeneity and were both more spread across the CCA2 axis, which was positively correlated with both a value for management decisions which were supported by scientific advice, as well as those which were cost-effective. The second component was negatively correlated with farmers who saw nature’s primary value as a home for wildlife. The management inputs most effected by attitudes were herbicide usage, use of other vegetation control, volume and cost of herbicides used per hectare, and selling of OP to a wholesaler. See S2 Table for highest factor loadings for dependent attitudinal factors, and S3 Table for descriptive statistics of the interaction between all questionnaire responses.

### Malaysian study sites

#### Summary of results

As in Indonesia, the extent of heterogeneity within and between farmer typologies varied between survey sections. Independent farmers were the most homogenous group in socio-demographic and attitudinal survey sections, while WAGS-BIO were the most heterogenous group across all three survey sections. Socio-demographics effected attitudes, and attitudes effected management inputs, with the most heterogeneity again seen in WAGS-BIO farmers. Economic factors, district, farmer age, and education level had the most impact on farmer attitudes, while attitudes towards pests and prioritization of yields most affected management inputs.

### Average sociodemographics of Malaysian farmers

**Fig 7:**
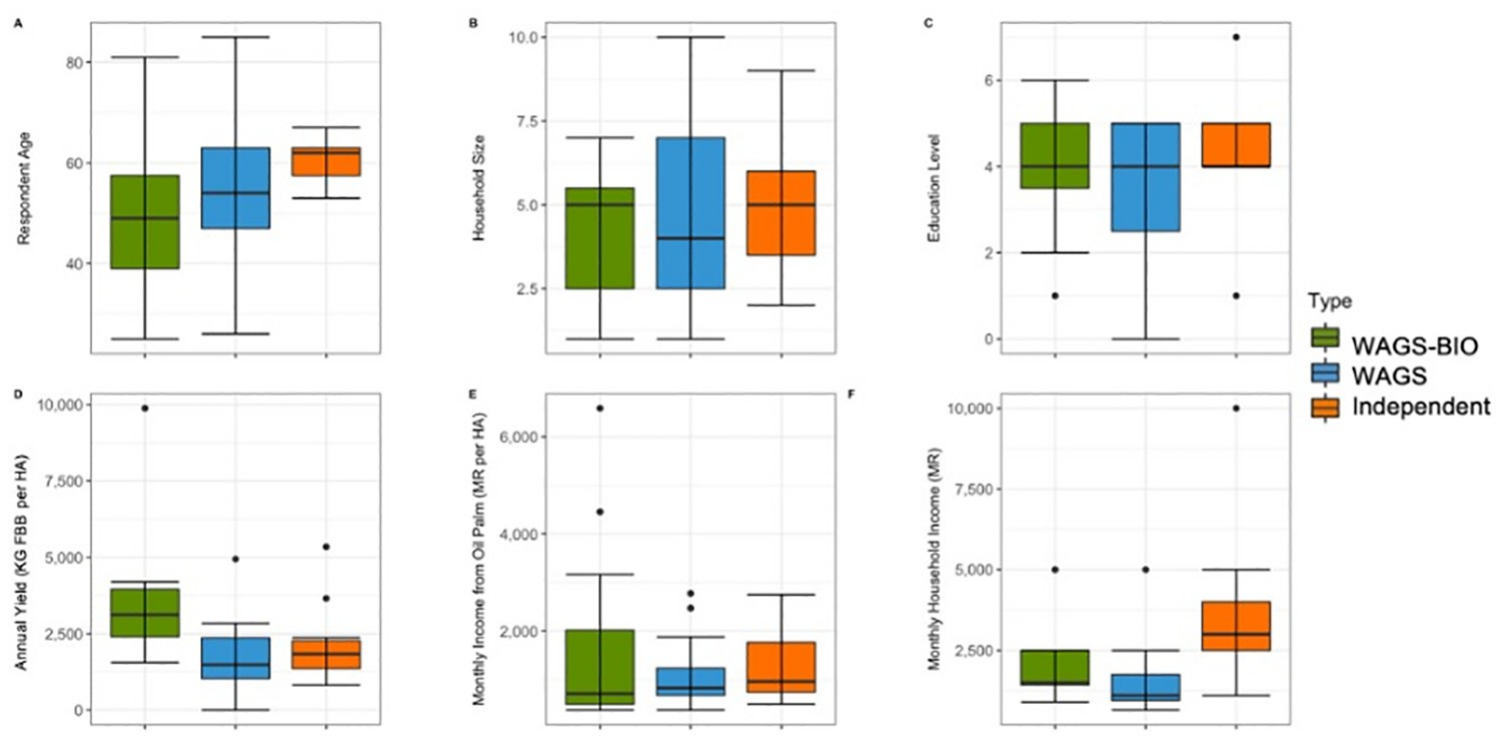
Comparisons of Malaysian farmer responses for the Socio-Demographic questions with numerical responses (A-F). Median, interquartile range, range, and outliers are given.

The average age of respondents was lowest for WAGS-BIO farmers (Average = 48.3, SE = 3.7), and highest for Independent farmers (Average = 60.4, SE = 1.1) (Fig 7A). The age of farmers was most homogeneous within the Independent farmers group. The average household size was similar for the three farmer typologies (WAGS-BIO: Average = 4.3, SE = 0.5; WAGS: Average = 4.8, SE = 0.6; Independent farmers: Average = 4.9, SE = 0.5), with the largest household seen in a WAGS farm (10 people) (Fig 7B). The average education level was the same across groups (SPM), with the lowest level of education only in WAGS farms (No formal education), and the highest level (Degree) only in an Independent farmer’s farm (Figs 4 and 7C). Average annual yield in kilograms per hectare was similar for WAGS and Independent farms (WAGS: Average = 1705.4, SE = 263.7; Independent farmers: Average = 2078.8, SE = 291.1), and highest in WAGS-BIO farms (Average = 3433.9, SE = 512.7) (Fig 7D). The highest yield of any farmer was seen in a WAGS-BIO farm (10000 KG), nearly four times the average across all study sites. The amount of variation in yield was similar for all three farmer typologies. Average monthly income from oil palm per hectare was similar across all farmer typologies (WAGS-BIO: Average = 1118.1, SE = 471.7; WAGS: Average = 1139.2, SE = 160.1; Independent farmers: Average = 1264, SE = 298.6), with the greatest variation and high outliers in WAGS-BIO (Fig 7E). Average total household income from all sources was highest in Independent farmers (Average = 3573.3, SE = 547.4), nearly twice that of WAGS farmers (Average = 1565.8, SE = 234.9), which was slightly below that of WAGS-BIO farmers (Average = 2080, SE = 335.5). The range of average income was greatest in Independent farmers, with one particularly high outlier (10000 MR) (Fig 7F).

#### Average management inputs of Malaysian farmers

**Fig 8:**
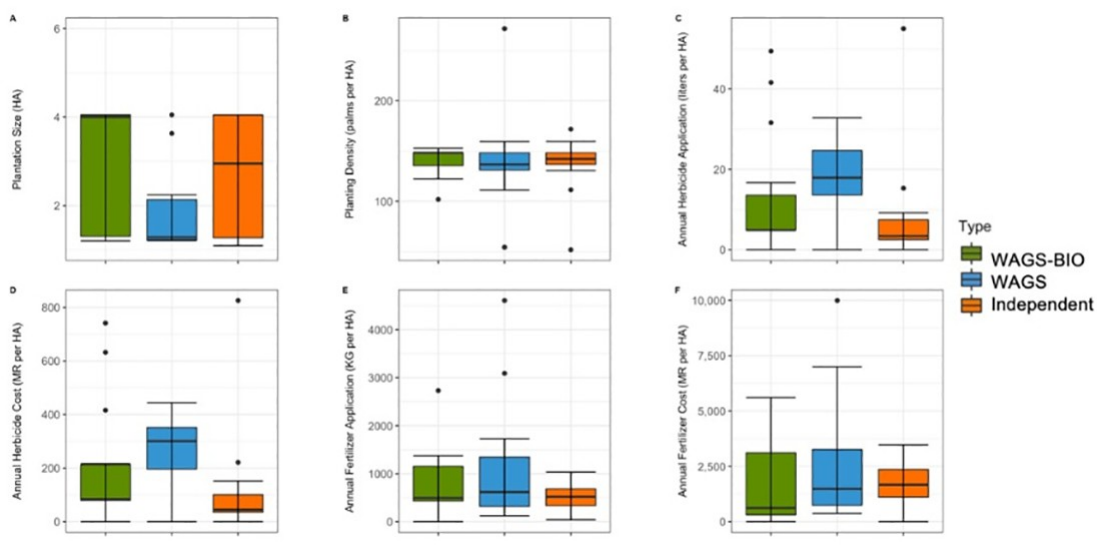
Comparisons of Malaysian farmer responses for the Management Input questions with numerical responses (A-F). Median, interquartile range, range, and outliers are given.

The average plantation size was largest in WAGS-BIO farms (Average = 2.38, SE = 0.41), and smallest in WAGS farms (Average = 1.35, SE = 0.23) (Fig 8A). The variation in average plantation size was similar for WAGS-BIO and Independent farms (Average = 2.69, SE = 0.34), and more homogenous in WAGS farms. Palm planting density was similar across all three farmer typologies in terms of average and range (WAGS-BIO: Average = 140.6, SE = 3.5; WAGS: Average = 138.2, SE = 10; Independent farmers: Average = 137.5, SE = 7) (Fig 8B). The average amount of herbicide used annually was highest in WAGS farms (Average = 18, SE = 2.2), and lowest in Independent farms (Average = 7.9, SE = 3.5), with the greatest number of outliers in WAGS-BIO farms (Average = 12.4, SE = 4) (Fig 8C). Similarly, the average herbicide cost per hectare was greatest in WAGS (Average = 265.9, SE = 30.1), and lowest in Independent farms (Average = 117, SE = 52.9) (c 8D). The average fertiliser amount used per hectare was highest in WAGS farms (Average = 1045.3, SE = 260.3), nearly twice that of Independent farmers (Average = 521.9, SE = 74.3), with outliers seen in WAGS farms and the least variation seen within Independent farms (Fig 8E). The average fertiliser cost followed a similar pattern in response (WAGS-BIO: Average = 1756.5, SE = 487.7; WAGS: Average = 2439.8, SE = 568.8; Independent farmers: Average = 1643.6, SE = 260) (Fig 8F).

#### Independent factors between site types

**Fig 9:**
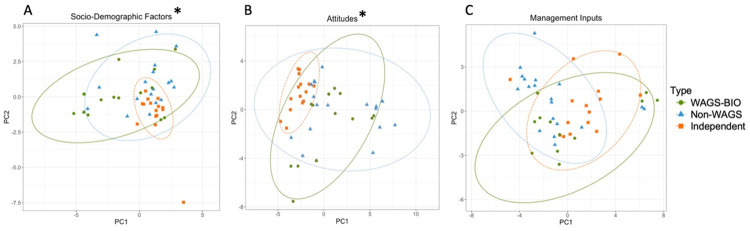
Results of Principal Component Analysis, showing variation in questionnaire answers for socio-demographic (A) farmer attitude (B), and management input (C) factors in Malaysia. Points are spherically grouped by farmer typology for the three different farmer typologies. The dotted line grouping each Farmer Typology is based on a 95% confidence interval. The Asterisk (*) represents statistically significant models (p > 0.05). Note: Scale differs between figures.

For socio-demographic factors, the first component accounted for 23% of variation and the second component for 19%. There was a statistically significant difference between farmer typologies (ANOSIM R = 0.1719, p = 0.0003), but large overlap between some groups, with socio-demographics of Independent farmers fully within WAGs and WAGS-BIO ellipses (Fig 9A). The highest heterogeneity was amongst WAGS-BIO farmers, particularly along PC1, which was most positively correlated with respondents being landowners and being involved in additional non-agriculture industries, and negatively correlated with farmers who were married. Independent farmers were the most homogenous group, particularly across the PC2 axis, indicating their positive correlation with the district BP and negative correlation with total household income and marital status. WAGS farmers were pulled more evenly along both axes (S4 Table).

For attitudinal factors, the first component accounted for 24% of variation, and the second component for 11%. There was a statistically significant difference between farmer typologies (ANOSIM R = 0.2038, p = 0.0001), although not all groupings were distinct, with high levels of overlap between groups, particularly for Independent farmers, which were within the ellipses of the other farmer groups. Again, there were higher levels of homogeneity in Independent farmers, who showed low variation in response across PC1, and lower variation than the other two farmer typologies across PC2 (Fig 9B). WAGS-BIO and WAGS farmers were much more heterogenous in attitudes along both axes, with WAGS farmers being more varied along PC1, which was most strongly negatively correlated with a higher regard for the aesthetic, economic, and cultural value of nature. WAGS-BIO farmers were more varied along PC2, which was positively correlated with farmers who reported themselves as making management decisions based on striving for consistency, and negatively correlated with a tendency to determine herbicide application timing by direct observation of weeds, rather than habit or instruction from external sources (S4 Table).

For management inputs, the first component accounted for 23% of variation, and the second for 10%. There was no significant difference between farmer typologies (ANOSIM R = 0.0448, p = 0.075), and farmer groups were not distinct, with overlap along both axes. WAGS-BIO showed the greatest heterogeneity, with greater variation across PC1, which was most positively correlated with no herbicide use and use of non-chemical methods of vegetation clearing, and negatively correlated with higher frequency of herbicide application and great amounts of herbicide applied. WAGS and Independent farmers showed similar levels of variation and opposing skew across PC1. WAGS farmers were the group most pulled along PC2, which was positively correlated with herbicide-free farming, and negatively correlated with other methods of vegetation clearing, in particular cut and drop vegetation clearing (S4 Table).

#### Relationships between factors by site type

**Fig 10:**
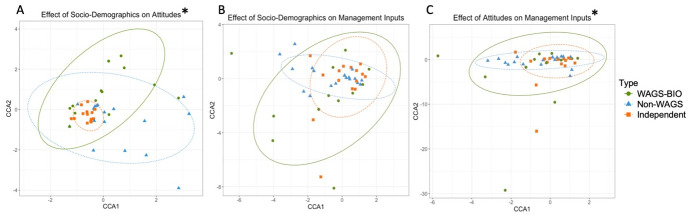
Results of Canonical Correspondence Analysis, showing the relationship between farmer responses on socio-demographics to attitudes (A), socio-demographics to management inputs (B), and attitudes to management inputs (C) in Malaysia. Points are spherically grouped by farmer typology for the three different farmer typologies. The dotted line grouping each Farmer Typology is based on a 95% confidence interval. The Asterisk (*) represents statistically significant models (p > 0.05).

Farmer socio-demographic factors had a significant effect on attitude (F = 1.98, p = 0.001). The first component explained 25% of variation, while the second component explained 14% of variation. Independent farmers were by far the most heterogenous group and fully overlapped with the other two farmer types (Fig 10A). WAGS-BIO farmers showed greater variation in CCA2, while WAGS showed more variation in CCA1 with several outliers. The first component was significantly positively correlated with District BP (F = 3.22, p = 0.001), District K (F = 4.10, p = 0.004), and formal education finishing at Form 2 (F = 3.039, p = 0.020). It was negatively correlated with the percentage of household income from agriculture (F = 2.57, p = 0.003), and higher total household income (F = 2.12, p = 0.003). The second component was most positively correlated with farmer age (F = 3.16, p = 0.001), and landowner status (F = 2.69, p = 0.005). The attitudes which were most effected by socio-demographics were the ‘most welcome’ wildlife in the plantation (particularly feral dogs, rats, long tailed macaques, and cobras), as well as farmer attitudes towards wildlife. See S5 Table for full table of highest factor loadings for dependent attitudinal factors and S6 Table for descriptive statistics of the interaction between all questionnaire responses.

Socio-demographic factors did not have a significant effect on management inputs (F = 1.32 p = 0.12). The first component explained 63% of variation, while the second component explained 19% of variation. WAGS-BIO farmers were more varied than the other groups along both axes, and completely overlapped with both WAGs and Independent farmers (Fig 6B). All three groups had outliers. Independent farmers varied equally across both axes, whereas WAGS farmers varied more along CCA1. The first component was non-significantly positively correlated with land owned by the government and District K (S5 Table). It was significantly negatively correlated with a lack of formal education (F = 4.76 p = 0.017). The second component was positively correlated with a lack of formal education and government land ownership, and negatively correlated with District HP (S5 Table). The management input factors most effected by socio-demographic factors were fertiliser volume per hectare, location of herbicide spraying, method of wildlife prevention, intercropping decisions, and use of organic manure. See S5 Table for full table of highest factor loadings for dependent attitudinal factors and S6 Table for descriptive statistics of the interaction between all questionnaire responses.

Attitudes had a significant effect on management inputs (F = 1.46, p = 0.033), with the first component explaining 69% of variation, and the second component explaining 14% of variation. There was substantial overlap between groups, with both Independent farmers and WAGS encompassed by WAGS-BIO ellipses, which showed greater variation than the other typologies along both axes (Fig 10C). All three farmer typologies were more varied along CCA1 than CCA2, particularly WAGS, and all groups had outliers. The first component was positively correlated with farmers who made their decisions based predominantly on yield (F = 4.29, p = 0.019), and who made decisions on chemical treatment based on direct observation of pests, rather than season or advice (F = 2.69, p = 0.48) (S5 Table). It was most negatively correlated with farmers who had the most negative attitudes to bagworm caterpillars in the plantation (F = 3.35, p = 0.017), rather than dangerous wildlife. The second component was most negatively correlated with farmers who view the main value of nature to be economic. The management inputs most effected by farmer attitudes were use of poison as a wildlife prevention, intercropping decisions, location of herbicide spraying, livestock presence, and use of grass machines for vegetation clearing. See S5 Table for full table of highest factor loadings for dependent attitudinal factors and S6 Table for descriptive statistics of the interaction between all questionnaire responses.

## Discussion

### Summary and background on management-assistance programs

We found that smallholder farmers are an incredibly diverse group, and this variability requires further study. The most significant independent and dependent factors varied across study section and country, but herbicide usage, vegetation clearing, village/district, and socio-economic factors were consistently prominent in distinguishing farmer groupings. While only socio-demographics significantly affected management inputs in Indonesia, attitudes significantly affected management inputs in Malaysia, indicating the importance of tailored recruitment and education initiatives to support smallholders in sustainable cultivation practices. The most-varied farmer typology for the two locations were at polar ends of the spectrum of degree of partnership: in Indonesia, Independent farmers free from a relationship with industry often displayed the highest variation in response, whereas in Malaysia the most tightly organized group (WAGS-BIO) were the most heterogeneous.

This apparent inconsistency may be due to differences in the way partnerships are organized when led by the private sector (as by SMARTRI in Indonesia), versus non-governmental programs (as by Wild Asia in Malaysia). From the initial establishment of the program there are clear differences: SMARTRI’s smallholder program is mandated by the Indonesian government, which stipulates that industrial palm oil producers, such as PT SMART Tbk, must have 20% representation from smallholders in their total land area managed. Therefore, smallholder farmers were not recruited by SMARTRI, but rather led into the program by Indonesian government officials at a district level. In contrast, Wild Asia’s WAGS program was developed out of an independent desire of the non-profit social enterprise to “support healthy ecosystems and bolster local livelihoods”, with farmers recruited by word of mouth and outreach programs. This difference in mandatory versus voluntary involvement may mean that the subset of farmers participating on their own accord are potentially more engaged and curious about management techniques, however they are also likely engaged with a range of ideas, which could explain the heterogeneity we identified. In addition, the farmers in SMARTRI programs have been involved with management-assistance since the onset of their plantation, whereas farmers in Wild Asia programs are able to begin their relationship with the management-assistance program at any time, and therefore have received different influence at different phases of their plantation lifecycle. This difference in program development and structure likely has large effects on style of interaction with smallholders and strictness of adherence to management recommendations by farmers.

In government-mandated private sector management-assistance schemes, such as SMARTRI, Fully Managed smallholder plantations have designated plantation managers and multiple plantation assistants responsible for applying inputs to their plantations. The plantation managers treat groups of smallholder farms under the same management regime as the larger industrial plantations, thereby largely removing the act of “farming” from a “smallholder farmer’s” day. In the public sector, however, there are less labour and financial resources, and regimes are often suggested and supported, rather than being directly implemented. For example, in the WAGS-BIO program smallholders are given information on the importance of soil nutrients and given direct demonstrations of mechanical and chemical input application. Therefore, the participant is still ‘farming’ and applying inputs themselves, likely leading to more variation in how consistently regimes are implemented.

### Variation in responses by survey-section

#### Socio-demographics

Indonesian Fully Managed farmers (those most closely tied to the industrial plantation), displayed the highest variation in responses, indicating that there is no ‘typical’ smallholder who participates in such schemes. Furthermore, there was large overlap between farmer typologies, and the averages of factors regarded as crucial to smallholder systems [64], such as education level, age, and household size, did not vary greatly between typologies. While previous research has reported distinct differences in farmers who participate in climate smart agriculture [65], or adapt to new farming technology [66], our findings suggest that SMARTRI schemes are not necessarily attracting a specific farmer demographic, and have the potential to interact with broader and more demographically diverse communities. As farmer involvement is determined by governments at a regional level and there is no recruitment process by SMARTRI, and therefore no bias towards a particular socio-demographic, this is unsurprising. Partially Managed and Independent farmers were skewed largely along the axis correlated with farmer involvement in an industry other than agriculture and total household income, and negatively correlated with the proportion of total household income that was derived from agriculture. This indicates that socio-economic factors, rather than educational or cultural factors, may be more influential in determining smallholder participation, mirroring results from research on participation in Agri-environmental schemes [67]. As similar management-assistance schemes have been shown to increase household income [68], this finding also suggests that advertising the income benefits of involvement could be effective in attracting new participants.

In Malaysia, Independent farmers (those least closely tied to Wild Asia) displayed the least variation in responses. This may be due to the close proximity of households within each typography, meaning they are likely to share the same socio-economic class, or may even be related, and therefore have similar opportunities for other employment, and sites may share land use history. The tight grouping of Independent farmers in socio-demographics suggests that a certain type of smallholder is attracted to volunteer to participate in NGO assistance schemes, in contrast to our findings from private sector partnerships in Indonesia, where government programs effectively determine participation. As socio-economic factors were the most impactful in ordinations, and the average income was highest in Independent farmers, this suggests that perhaps high-income smallholders are less attracted to NGO partnership schemes, perhaps because potential benefits are most marked for lower-income groups. For higher-income groups, this lack of participation may therefore be due to a lack of apparent financial benefit or because farms already possess a lucrative cultivation system. While programs can bring economic benefits for smallholders, they can also keep farmers bound to a specific market and not able to take full advantage of market competition or the range of inputs available [69]. Organizations which are ecologically-focused, could therefore prioritize marketing to recruit environmentally minded, lower income farmers, spread across a wider geography.

In both locations, income and socio-economic factors had the strongest relationship with degree of partnership. Across Southeast Asia, oil palm has historically been farmed as a profit-driven crop and lacks the cultural or sustenance background it has in its native West Africa, where it is associated with more traditional uses [70]. Therefore, within Southeast Asia, our results indicate that the potential price premium generated by certified palm oil [71] and other financial incentives are likely to be highly relevant for attracting smallholder participation in partnership schemes and increasing awareness. As income was more variable than other socio-demographics in both regions, it is also likely that smallholders have different lived experiences due to financial status. This highlights the importance of schemes contributing to farmer livelihoods, to ensure social sustainability of oil palm cultivation [16]. Indeed, “Decent Living Wage” has recently been added to the Principles and Criteria of RSPO guidelines, highlighting the importance of this factor [72]. Further research on the financial benefits of participating in smallholder schemes is required to determine if income level is a reason for, or result of, involvement in management-assistance programs.

The average yields reported for organized farmers in both Indonesia (11.6 tFFB ha^−1^ yr^−1^) and Malaysia (13.5 tFFB ha^−1^ yr^−1^) were lower than standard reported yields in nucleus and former nucleus plantations (17–22 tFFB ha^−1^ yr^−1^) [33, 73]. However, there was a large difference in average yields for independent farmers in Indonesia (6.2 tFFB ha^−1^ yr^−1^) and Malaysia (15.1 tFFB ha^−1^ yr^−1^) which differed substantially from previous findings for independent plantations (10 tFFB ha^−1^ yr^−1^) [17]. While the wide range in reported values does not necessarily mean that these are inaccurate, we nevertheless call for capacity building to improve smallholder record keeping, and independent verification of reporting by mills and partner programs (see ‘Caveats to Study”).

#### Attitudes

In Indonesia, farmer typologies were most distinct from one another in the attitudes survey section, with Independent farmers being particularly distinct. As it is unclear from our study set-up whether this is due to attitudes determining a farmer’s likelihood to be involved in partnership schemes offered by local governments, or involvement in a partnership scheme changing farmer attitudes. Further research, potentially employing a BACI experimental design [74], is required. This distinction between typologies, which was not present in the socio-demographic survey section, suggests that it is externally-manipulable factors such as attitudes, rather than more ingrained factors such as socio-demographics, that determine involvement. In particular, attitude responses that are potentially most influenced by the private sector, including access to scientific advice and cost of inputs, had the biggest impact on distinction of typologies, and are therefore particularly relevant for local partnership schemes, and could be targeted to increase engagement.

In Malaysia, there was a large overlap in attitudes, and Independent farmers were again the most heterogenous group. This heterogeneity may again be due to the close geographic proximity of sites, particularly in Independent farmers, as local communities often share social norms and attitudes through peer relationships [75]. As socio-demographics and attitudes were particularly heterogenous in Independent farmers, it could be that approaching new geographies with targeted outreach could attract a new participant pool. In these areas, further surveys should question why farmers have chosen to remain independent, and specifically whether this choice was due to access to information about schemes, or perceived value of involvement. Farmer attitudes towards the value of nature were most strongly related to degree of partnership in Malaysian sites, which is likely related to Wild Asia being an ecologically-focused NGO, and thus highlights the value of the programme for supporting conservation. Furthermore, this is likely to promote a feedback cycle between the initial farmer involvement, WAGS and WAGS-BIO program education, and farmer attitudes towards nature. To learn more from participating smallholders, further research could investigate why smallholders elected to participate in schemes in the first instance, and how satisfied they are with the outcomes of their participation. While such detailed open questions were not appropriate in this initial scoping survey, further satisfaction surveys of partnered smallholders would be beneficial to direct future strategies and initiatives, and development of the program. As WAGS-BIO farmers have specifically chosen to develop their status from WAGS to WAGS-BIO, it is likely they have a positive outlook on the program.

While Indonesian and Malaysian sites differed in degree of overlap between groups and most strongly influential attitudes, both locations would benefit from more detailed interviews on the effects of partnership on attitudes through a BACI design survey, and on the satisfaction of farmers with their respective management-assistance programs.

#### Management inputs

The three Indonesian farmer typographies were least distinct in the management input survey section. This is an intriguing finding, as management inputs represent the aspect where smallholders are most directly involved with SMARTRI. This suggests that Partially Managed farms are adhering strictly to the suggested regime and are therefore very much in line with the inputs directly applied by plantation managers in Fully Managed farms, and that the practices of Independent smallholders are not from those of plasma estates. Alternatively, it could be that smallholders were reporting values based off memory rather than record, and simply repeating input levels that have been suggested to them. Again, we suggest improved capacity-building for farmer record-keeping, and increased verification of input levels, which could help to verify our findings. The large heterogeneity in responses of Independent farmers on management inputs highlights the high variability in management practices amongst this group. While it is unclear which inputs are most influential in determining the higher average yield reported in Fully and Partially Managed farms in this study, previous research has found that the key factors contributing to high yield in certified sustainable oil palm plantations are quality of seedlings and increased fertiliser application [10, 50], the latter of which was highest in Fully Managed farms. Interestingly, despite the higher fertiliser application in Managed farms the cost of fertiliser does not show significant differences. This may be due to a higher purchasing price of fertilisers per unit for Independent farmers, as they have less leverage to negotiate selling price with fertiliser suppliers, while Fully Managed farmers have access to economies-of-scale prices.

In Malaysia, there was less homogeneity than expected in WAGS-BIO farmers, considering these farmers are on a set management plan. This is likely due to the fact that the farmers in the WAGS-BIO grouping were at different phases of their journey with WAGS-BIO; some farmers had been under the instructed management regime for more than a year, while some farmers had not yet gone through a full growing season under the chemical free regime. While sensitivity analysis showed that the duration of involvement in WAGS-BIO did not have a statistically significant effect on any survey section, it still may be responsible for visual differences in PCA analysis. Also, as the program inputs are not applied directly by Wild Asia, in contrast to the Indonesian program, this variation could be because farmers are treating the program as an additional source of information rather than following guidelines strictly, and therefore suggestions from the WAGS-BIO program may be conflicting with recommendations from other non-organic extension services. For example, Malaysian public-sector extension services are accessed by nearly 250,000 oil palm smallholders working with over 300 extension officers working for the Malaysian Palm Oil Board [76], which suggests a certain level of herbicide application. In particular, WAGS-BIO farmers are encouraged by Wild Asia to use no herbicide, but reported higher average herbicide usage than Independent farmers. It is not uncommon for certified smallholders to not fully-follow certification guidelines [14], perhaps explaining this discrepancy. As values are self-reported, this could also be due to accidental misrepresentation of values in surveys. Regardless, further work is needed to verify farmer practices and improve farmer record keeping capacity.

Both Malaysian and Indonesian independent farmers receive information from numerous sources, such as neighbours, media, and extension systems [77, 78]. They therefore experience a more varied influence, than the non-independent farmers, that will contribute to determining their realised management system, as seen in their increased heterogeneity in Malaysia. For many farmers, cultivation of oil palm is a “first generation” activity, resulting in a lack of heritage knowledge in oil palm not seen in crops with longer cultivation history. Thus, farmers are less aware of good agricultural practice and in greater need of outside information. Not being part of an organized regime, Independent farmers are free to alter their management inputs based on weekly, monthly, or quarterly changes in circumstances. These circumstances may be personal, such as finances or labour availability [79], or shared across a community or region, such as weather and pest infestations [11]. In recent years, price of palm oil has been a particularly influential factor for oil palm farmers, with the price of crude palm oil varying between USD$530 to USD$1000 over the last five years [80]. This variation can be highly influential in determining the appeal of oil palm agriculture compared to other crops, as well as determining the funds independent smallholders have available for inputs.

In both countries, decisions regarding vegetation management showed the strongest factor loadings, suggesting that this is a key area for both the private and public sector to engage smallholders. Vegetation management varies greatly between plantations [52], and maintenance of understory vegetation can help support plantation biodiversity [53], soil health [81], decomposition [51], and pest abundance [82]. The continued importance of vegetation management in our surveys show that herbicide usage and understory clearing are important areas to influence smallholders, particularly as increased clearing may not necessarily contribute to higher yields. In our study, Independent farms had the lowest average yield, and highest herbicide application (nearly 2x more than Fully Managed farmers). While the value of understory vegetation has been well documented, a large proportion of smallholders continue to practice complete clearing [14], indicating that this is a key area for assistance programs to target with increased training. SMARTRI’s management regimes emphasize the importance of maintaining a high level of vegetation ground cover in the understory, which was reflected in quantity of herbicides used in Fully Managed farms, and to some extent in Partially Managed farms compared to Independent farms.

### Interaction between responses by survey-section

#### The effect of socio-demographics on attitudes

In Indonesian sites, socio-demographics did not influence attitudes, potentially due to the high level of heterogeneity in both variables. In contrast, in Malaysian sites socio-demographics significantly affected attitudes, with markedly more variability in the two organized smallholder typologies (WAGS-BIO and WAGS), than in Independent farmers, indicating that these farmers may share more viewpoints with those ‘similar’ demographically, and therefore participate in such programs. In both countries, district/village heavily influenced attitudes, indicating that while farmers may not report that they rely on their neighbours for guidance and information, community-bonds may still be influencing viewpoints and priorities. Income from agriculture and total income influenced attitudes, again indicating that socio-economics may be a key determinant of involvement, and an area for partnership-programs to emphasise during recruitment.

#### The effect of socio-demographics on management inputs

In Indonesia, socio-demographics had a significant effect on management inputs, with the most influence from village/district, household size, and income. Again, the significance of village suggests that there may be knowledge sharing within communities, potentially passively, through observation and unspoken societal norms, or actively, through workshops and demonstrations [83]. Knowledge sharing programs that make use of community links, through practices such as community demonstrations, may therefore prove a fruitful avenue to capitalize on these bonds, and ensure shared practice is best practice. In Malaysia, socio-demographics did not have a significant effect on management inputs.

Income, which was a significant factor in Indonesia and had a high factor loading in Malaysia, plays a significant role in determining management. This is likely to be because smallholder farmers are constrained in their management inputs by what seeds, fertilisers and herbicides they can afford [84]. This is likely to have a more marked impact on independent farmers, the most heterogenous group, who pay for their own inputs. In both contexts, herbicide usage and vegetation control were responses most affected by socio-demographics, further strengthening the argument that these are among the most important factors for sustainability organizations to target. Similarly, across contexts, socio-demographics impacted organic fertiliser usage, which is known to influence greenhouse gas emissions [85], soil fertility [86], and yield [87]. As fertiliser is a major limiting factor for yield [10] and its availability is expected to decrease in the future [88], supporting the use of organic, alternative materials as fertilisers is a potentially important area for engagement.

#### The effect of attitudes on management inputs

In Indonesia, respondent attitudes did not have a significant effect on management inputs. As socio-demographics *did* have a significant effect on management inputs, this indicates that it may not be opinions and beliefs, but rather more constraining factors (such as socio-economic factors) that most affect management decisions. As many partnership programs, including those in this project, involve both educational aspects and direct benefits to farmer socio-economics, this may indicate that programs should focus on increasing farmer awareness of the socio-economic benefits of involvement, rather than aiming to alter attitudes. Again, the management inputs most effected by attitudes were related to herbicide usage and vegetation control, highlighting the likely influence of economic factors.

In contrast, in Malaysia there was a significant effect of attitudes on management inputs, with the highest variability in WAGS-BIO, the most organized group. This finding could help direct future research and engagement by Wild Asia; as prioritisation of yields and attitudes towards pests were the most significant factors identified, programs which focus on informing farmers about the potential benefits of involvement related to these factors could be most effective. The focus on yield, recorded here and in other sections, logically coincides with a high motivation by smallholders to control pests. This also explains the predominant negative attitude towards bagworm caterpillars, which are well-known pests of oil palm and can heavily reduce yield [89]. This suggests, once again, that oil palm smallholders are primarily focused on yield, which should be respected when suggesting management inputs and plans.

The variation between factors that influence management inputs in Indonesia and Malaysia exemplifies the variability in smallholder priorities and influences, validating the importance of a tailored approach to smallholder groups. Programs aimed at improving smallholder productivity and sustainability must consider the heterogeneity of the smallholder oil palm sector. Although our study faced several limitations, we believe it contributes to a better understanding of the challenges and opportunities within the smallholder sectors of the oil palm industry.

### Caveats to study

In research and in practice, it is crucial to account for the heterogeneity of the smallholder oil palm sector to avoid ineffective and inconsiderate one-size-fits-all solutions. While our research illuminates a snapshot of the variety of smallholders, findings are implicitly only relevant to smallholders directly involved in our study, and further research is needed to catalogue the full variability in socio-demographics, attitudes, and management approaches. This is therefore both a limitation, and an important finding, of our study.

Surveys were conducted when government COVID-19 travel restriction mandates were still in place. This restricted the ability for researchers to travel between Indonesia and Malaysia, reducing comparability between sites and resulting in several questions which were approached differently in different sites. Moreover, reduced travel meant that we had limited geographical spread, and therefore were unable to choose sites with an ideal study layout. As such, village and district dynamics were potentially more influential in our results as communities were close, as outlined across the discussion.

Our results are based on a self-reported survey, with the potential for misreporting by respondents. In particular, smallholders often do not maintain consistent and accurate records of yields [18], and their estimations may have been over-optimistic, or over-pessimistic. Although uncertainties are inherent in working with data collected through interviews, we believe the use of ordinations limited the potential of singular outliers to heavily skew our data. We also call for improved record keeping and verification of inputs by partner organizations, as only though accurate recording of yield will we be able to identify effective and realistic management programs.

## Conclusion and implications

Our study aimed to determine the patterns and variety in socio-demographics, attitudes, and management inputs across smallholders of varying partnership with outside organizations. We found that smallholders, independent and organized, are an incredibly varied group, with heterogeneity across all survey sections. This variability provides increased opportunity for sustainability schemes to operate in this space, as there is likely a myriad of smallholder farmer typologies to be found. Thus, there is the potential to identify existing smallholders whose practices are already high-yielding and biodiversity-friendly, informing the development of sustainability guidelines and requiring further study. Conversely, there is likely a large number of smallholder farmers currently operating in low-yield, environmentally-unfriendly systems, who require greater engagement and attention. This variation also indicated that socio-demographics and attitudes require context-specific programs to effectively impact and support smallholder farmers. We call for increased representation of smallholder farmers during the development of partnership schemes, to ensure that a diversity of local perspectives and demographics are represented. However, while it is important to have high representation of smallholders, it seems that the potential for ecologically-sustainable plantations depends on there being a high level of agroecological knowledge amongst farmers, which does not yet appear to have been acquired by many of these smallholders in both countries. Therefore, this call relies on increased access to extension services.

While our investigation used case studies and is therefore not applicable to oil palm smallholders across Southeast Asia, we recorded distinct trends in smallholder groups. We found that socio-demographics and attitudes can influence management inputs, albeit in varying degrees and directions, and therefore provide avenues to influence farmer practices. We have also consistently recorded the central role that vegetation management plays in decisions and suggest this as a key area for further study. While this was the first social scoping exercise run at these sites, our study demonstrates the value of such studies to identify groups who it may be most effective to work with, and that approaches such as this can reveal methods to approach groups most effectively. Further research, including BACI designed surveys, is needed to identify the direction of relationships between factors and to determine whether trends are consistent across regions, partnerships systems, and scales.

## Supporting information

S1 Text Data availability statement. (DOCX)

S1 Table PCA descriptive statistics for Indonesian sites. Principal Component Analysis (PCA) descriptive statistics for Axes 1 and 2 of all questionnaire responses in Indonesian sites.
(DOCX)

S2 Table CCA factor loadings for Indonesian sites. Full table of highest Canonical Correspondence Analysis (CCA) factor loadings for dependent factors in Indonesian sites.
(DOCX)

S3 Table CCA descriptive statistics for Indonesian sites. Canonical Correspondence Analysis (CCA) descriptive statistics for the interaction between all questionnaire responses in Indonesian sites. An asterisk (*) represents statistically significant factors (p > 0.05).
(DOCX)

S4 Table PCA descriptive statistics for Malaysian sites. Principal Component Analysis (PCA) descriptive statistics for Axes 1 and 2 of all questionnaire responses in Malaysian sites.
(DOCX)

S5 Table CCA factor loadings for Malaysian sites. Full table of highest Canonical Correspondence Analysis (CCA) factor loadings for dependent factors in Malaysian sites.
(DOCX)

S6 Table CCA descriptive statistics for Malaysian sites. Canonical Correspondence Analysis (CCA) descriptive statistics for the interaction between all questionnaire responses in Malaysian sites. An asterisk (*) represents statistically significant factors (p > 0.05).
(DOCX)

S1 File Inclusivity in global research. (DOCX)
