# Supplementary material for: Private, non-profit, and plantation: Oil palm smallholders in management-assistance programs vary in socio-demographics, attitudes, and management practices
Source: PLoS One. 2025 Jan 17;20(1):e0304837. doi: 10.1371/journal.pone.0304837 (PMC11741574; doi:10.1371/journal.pone.0304837)
Supplement: S1 Text — (DOCX) [file pone.0304837.s001.docx]

# Supporting Information

S1 Text: Data availability

Participant questionnaire uploaded to the University of Cambridge Data Repository; raw data uploaded to the Environmental Information Data Centre doi.org/10.5285/b61a12a2-d091-41af-b451-a14de4f4a3c3 and doi.org/10.5285/189164db-fe83-4b3f-b572-2fe99faba2d1
