# Supplementary material for: Private, non-profit, and plantation: Oil palm smallholders in management-assistance programs vary in socio-demographics, attitudes, and management practices
Source: PLoS One. 2025 Jan 17;20(1):e0304837. doi: 10.1371/journal.pone.0304837 (PMC11741574; doi:10.1371/journal.pone.0304837)
Supplement: S1 Table — Principal Component Analysis (PCA) descriptive statistics for Axes 1 and 2 of all questionnaire responses in Indonesian sites. (DOCX) [file pone.0304837.s002.docx]

**S1 Table. PCA descriptive statistics for Indonesian sites.** Principal Component Analysis (PCA) descriptive statistics for Axes 1 and 2 of all questionnaire responses in Indonesian sites.

| Factor | PC1 | PC2 |
| --- | --- | --- |
| Socio-demographics | | |
| Age | -0.163 | 0.322 |
| Household Size | -0.074 | 0.034 |
| Education Level | 0.200 | -0.261 |
| Percentage Income from Agriculture | -0.423 | -0.612 |
| Total Monthly Income | 0.255 | -0.087 |
| Monthly Income Per HA from Oil Palm | 0.089 | -0.087 |
| Years On Land | 0.031 | 0.034 |
| Village_BL | -0.074 | 0.055 |
| Village_BR | 0.152 | -0.021 |
| Village_GS | 0.139 | 0.038 |
| Village_KA | 0.046 | 0.046 |
| Village_KB | -0.104 | 0.046 |
| Village_KJ | 0.116 | 0.021 |
| Village_KM | 0.064 | -0.036 |
| Village_LJ | -0.130 | -0.025 |
| Village_PB | -0.066 | 0.204 |
| Village_PTP | 0.046 | -0.460 |
| Village_SB | -0.134 | 0.172 |
| Village_SL | 0.141 | 0.098 |
| Village_SM | -0.093 | 0.109 |
| Village_TS | -0.001 | 0.081 |
| Female | -0.204 | -0.482 |
| Male | 0.204 | 0.482 |
| No involvement in other industry | -0.471 | 0.013 |
| Attitudes | | |
| Importance of Nature_Economic | -0.107 | 0.048 |
| Importance of Nature_Food | 0.089 | -0.055 |
| Importance Nature_Wildlife | -0.104 | 0.068 |
| Importance Nature_Beauty | 0.017 | -0.180 |
| Importance of Nature_Culture | -0.014 | -0.155 |
| Importance of Nature_Health | 0.048 | -0.105 |
| Influence on Management _Neighbours | 0.174 | 0.148 |
| Influence on Management _Scientific | -0.237 | 0.073 |
| Influence on Management _Cost | 0.184 | 0.044 |
| Influence on Management _Effort | 0.114 | 0.084 |
| Influence on Management _Consistancy | 0.086 | 0.173 |
| Influence on Management _Yields | 0.081 | 0.178 |
| Preference for Agricultural Industry | -0.008 | 0.299 |
| Attitudes on Farming_Easy and sustainability | -0.043 | 0.225 |
| Attitudes on Farming_Economics | 0.043 | -0.225 |
| Herbicide Motivation_Weeds | 0.164 | 0.337 |
| Chemical Motivation_Pests | -0.296 | -0.110 |
| Fertilizer Type Motivation_Supplier | 0.349 | -0.094 |
| Fertilizer Type Motivation_Cooperative | -0.349 | 0.094 |
| Favourite Animal_Butterflies | -0.058 | 0.017 |
| Favourite Animal_Cobra | -0.102 | 0.053 |
| Favourite Animal_Dragonflies and damselflies | 0.135 | -0.095 |
| Favourite Animal_Leopard cat | -0.050 | -0.093 |
| Favourite Animal_Monitor lizard | 0.155 | -0.112 |
| Favourite Animal_Weaver ant | 0.017 | -0.190 |
| Favourite Animal_Yellow crazy ant | -0.058 | 0.055 |
| Least Favourite Animal_Bagworm caterpillar | -0.085 | 0.033 |
| Least Favourite Animal_Cobra | 0.044 | 0.211 |
| Least Favourite Animal_Long tailed macaque | -0.059 | 0.076 |
| Least Favourite Animal_Monitor lizard | 0.036 | 0.063 |
| Least Favourite Animal_N/A | -0.035 | 0.046 |
| Least Favourite Animal_Nettle caterpillar | 0.083 | 0.023 |
| Least Favourite Animal_Phyton | -0.057 | -0.050 |
| Least Favourite Animal_Rat | 0.045 | -0.170 |
| Least Favourite Animal_Rhinoceros beetle | -0.064 | 0.050 |
| Least Favourite Animal_Weaver ant | 0.012 | -0.130 |
| Least Favourite Animal_Wild pig | -0.064 | -0.065 |
| Management Inputs | | |
| Plantation Area | 0.124 | -0.084 |
| Palms Per Hectare | -0.086 | 0.025 |
| Hours Farming Weekly Per HA | 0.123 | 0.051 |
| Number of Herbicide Types | -0.132 | 0.166 |
| Herbicide Applications Annual | -0.056 | -0.114 |
| Herbicide Cost Annual | 0.185 | -0.285 |
| Herbicide Cost Per HA Annual | 0.193 | -0.288 |
| Herbicide Litres Annual | 0.215 | -0.278 |
| Herbicide Litres Per HA Annual | 0.228 | -0.279 |
| Number of Herbicide Methods | -0.069 | -0.151 |
| Number of Fertilizer Types | -0.189 | 0.019 |
| Fertilizer Cost Per HA Annual | -0.059 | -0.055 |
| Fertilizer Amount Per HA Annual | -0.160 | -0.057 |
| Number of OP Harvests Monthly | -0.265 | 0.044 |
| No Other Vegetation Control | 0.026 | 0.326 |
| Herbicide Location_Circle | -0.282 | -0.162 |
| Herbicide Location_Path | -0.282 | -0.162 |
| Herbicide Location_Multiple Methods | 0.282 | 0.162 |
| No Livestock Present | 0.067 | 0.020 |
| Livestock Present | -0.067 | -0.020 |
| No Organic Manure Use | -0.036 | 0.335 |
| Organic Manure Use | 0.036 | -0.335 |
| OP Buyer_PT. Sinarmas | -0.265 | 0.044 |
| OP Buyer_Wholesaler | 0.265 | -0.044 |
