# Supplementary material for: Private, non-profit, and plantation: Oil palm smallholders in management-assistance programs vary in socio-demographics, attitudes, and management practices
Source: PLoS One. 2025 Jan 17;20(1):e0304837. doi: 10.1371/journal.pone.0304837 (PMC11741574; doi:10.1371/journal.pone.0304837)
Supplement: S2 Table — Full table of highest Canonical Correspondence Analysis (CCA) factor loadings for dependent factors in Indonesian sites. (DOCX) [file pone.0304837.s003.docx]

**S2 Table: CCA factor loadings for Indonesian sites.** Full table of highest Canonical Correspondence Analysis (CCA) factor loadings for dependent factors in Indonesian sites.

| Factor | CCA1 | CCA2 |
| --- | --- | --- |
| Attitudes affected by socio-demographics | | |
| ImportanceNature_Economic | -0.036 | 0.045 |
| ImportanceNature_Food | -0.068 | 0.021 |
| ImportanceNature_Wildlife | -0.005 | 0.088 |
| ImportanceNature_Beauty | -0.095 | -0.059 |
| ImportanceNature_Culture | -0.115 | -0.058 |
| ImportanceNature_Health | -0.083 | -0.086 |
| ManagementInfluence_Neighbours | 0.185 | 0.008 |
| ManagementInfluence_Scientific | -0.060 | 0.079 |
| ManagementInfluence_Cost | 0.226 | -0.191 |
| ManagementInfluence_Effort | 0.051 | -0.089 |
| ManagementInfluence_Consistancy | 0.059 | 0.019 |
| ManagementInfluence_Yields | 0.078 | 0.038 |
| AgriculturalPreference_N | -1.281 | -0.930 |
| AgriculturalPreference_Y | 0.057 | 0.102 |
| HerbicideMotivation_Weeds_N | -0.426 | -0.217 |
| HerbicideMotivation_Weeds_Y | 0.333 | 0.265 |
| ChemicalMotivation_Pests_N | 0.327 | 0.088 |
| ChemicalMotivation_Pests_Y | -0.330 | -0.010 |
| FertilizerTypeMotivation_Supplier_N | -0.171 | 0.195 |
| FertilizerTypeMotivation_Supplier_Y | 0.261 | -0.297 |
| FertilizerTypeMotivation_Cooperative_N | 0.261 | -0.297 |
| FertilizerTypeMotivation_Cooperative_Y | -0.171 | 0.195 |
| FavoriteAnimal_Butterflies | 0.053 | -0.053 |
| FavoriteAnimal_Cobra | 0.598 | 0.210 |
| FavoriteAnimal_Dragonflies | 0.027 | -0.797 |
| FavoriteAnimal_Leopard | -4.473 | -0.367 |
| FavoriteAnimal_Monitor | -0.139 | -0.379 |
| FavoriteAnimal_N/A | 0.131 | 1.136 |
| FavoriteAnimal_Weaver | -0.086 | -0.525 |
| FavoriteAnimal_Yellow | -1.072 | 0.797 |
| LeastFavoriteAnimal_Bagworm | -0.450 | 0.549 |
| LeastFavoriteAnimal_Cobra | 0.209 | 1.228 |
| LeastFavoriteAnimal_Long | -0.675 | 2.634 |
| LeastFavoriteAnimal_Monitor | 0.938 | -0.575 |
| LeastFavoriteAnimal_N/A | -0.368 | 0.828 |
| LeastFavoriteAnimal_Nettle | 0.163 | 0.242 |
| LeastFavoriteAnimal_Phyton | -0.118 | -0.288 |
| LeastFavoriteAnimal_Rat | 0.038 | -0.209 |
| LeastFavoriteAnimal_Rhinoceros | 0.268 | -0.605 |
| LeastFavoriteAnimal_Weaver | -2.531 | -0.470 |
| LeastFavoriteAnimal_Wild | -0.520 | -1.434 |
| Management inputs affected by socio-demographics | | |
| PlantationArea | 0.197 | -0.166 |
| PalmsPerHectare | -0.043 | 0.025 |
| HoursFarmingWeeklyPerHA | -0.047 | 0.138 |
| NoHerbicideTypes | -0.160 | -0.127 |
| HerbicideApplicationsAnnual | 0.016 | 0.002 |
| HerbicideCostAnnual | 0.735 | -0.035 |
| HerbicideCostPerHAAnnual | 0.376 | 0.186 |
| HerbicideLitersAnnual | 0.844 | -0.046 |
| HerbicideLitersPerHAAnnual | 0.485 | 0.162 |
| NoHerbicideMethods | -0.056 | 0.045 |
| NoFertilizerTypes | -0.137 | -0.041 |
| FertilizerCostPerHAAnnual | -0.136 | -0.004 |
| FertilizerAmountPerHAAnnual | -0.100 | 0.046 |
| NoOPHarvestsMonthly | -0.161 | 0.032 |
| OtherVegetationControl_N | -0.078 | -0.250 |
| OtherVegetationControl_Y | -0.008 | 0.275 |
| HerbicideLocation_Circle_N | 0.407 | -0.023 |
| HerbicideLocation_Circle_Y | -0.122 | 0.033 |
| HerbicideLocation_Path_N | 0.407 | -0.023 |
| HerbicideLocation_Path_Y | -0.122 | 0.033 |
| HerbicideLocation_All_N | -0.122 | 0.033 |
| HerbicideLocation_All_Y | 0.407 | -0.023 |
| UseOfFronds_Stacked | -0.041 | 0.024 |
| LivestockPresence_N | -0.004 | 0.056 |
| LivestockPresence_Y | -0.111 | -0.035 |
| FertilizerUse_Y | -0.041 | 0.024 |
| OrganicManureUse_N | -0.138 | -0.084 |
| OrganicManureUse_Y | 0.497 | 0.626 |
| Intercropping_N | -0.041 | 0.024 |
| OPBuyer_PT. | -0.339 | 0.043 |
| OPBuyer_Wholesaler | 0.573 | -0.016 |
| Management inputs affected by attitudes | | |
| PlantationArea | 0.206 | -0.135 |
| PalmsPerHectare | -0.022 | -0.027 |
| HoursFarmingWeeklyPerHA | -0.050 | 0.049 |
| NoHerbicideTypes | -0.065 | -0.053 |
| HerbicideApplicationsAnnual | 0.028 | -0.075 |
| HerbicideCostAnnual | 0.706 | -0.034 |
| HerbicideCostPerHAAnnual | 0.391 | 0.170 |
| HerbicideLitersAnnual | 0.776 | 0.003 |
| HerbicideLitersPerHAAnnual | 0.463 | 0.196 |
| NoHerbicideMethods | 0.008 | -0.144 |
| NoFertilizerTypes | -0.084 | -0.084 |
| FertilizerCostPerHAAnnual | -0.132 | -0.003 |
| FertilizerAmountPerHAAnnual | -0.088 | 0.006 |
| NoOPHarvestsMonthly | -0.129 | -0.070 |
| OtherVegetationControl_N | 0.180 | 0.186 |
| OtherVegetationControl_Y | -0.199 | -0.207 |
| HerbicideLocation_Circle_N | 0.969 | 0.931 |
| HerbicideLocation_Circle_Y | -0.194 | -0.190 |
| HerbicideLocation_Path_N | 0.969 | 0.931 |
| HerbicideLocation_Path_Y | -0.194 | -0.190 |
| HerbicideLocation_All_N | -0.194 | -0.190 |
| HerbicideLocation_All_Y | 0.969 | 0.931 |
| UseOfFronds_Stacked | -0.017 | -0.019 |
| LivestockPresence_N | 0.047 | 0.066 |
| LivestockPresence_Y | -0.138 | -0.179 |
| FertilizerUse_Y | -0.017 | -0.019 |
| OrganicManureUse_N | -0.089 | -0.058 |
| OrganicManureUse_Y | 0.382 | 0.196 |
| Intercropping_N | -0.017 | -0.019 |
| OPBuyer_PT. | -0.294 | -0.146 |
| OPBuyer_Wholesaler | 0.554 | 0.242 |
