# Supplementary material for: Private, non-profit, and plantation: Oil palm smallholders in management-assistance programs vary in socio-demographics, attitudes, and management practices
Source: PLoS One. 2025 Jan 17;20(1):e0304837. doi: 10.1371/journal.pone.0304837 (PMC11741574; doi:10.1371/journal.pone.0304837)
Supplement: S3 Table — Canonical Correspondence Analysis (CCA) descriptive statistics for the interaction between all questionnaire responses in Indonesian sites. An asterisk (*) represents statistically significant factors (p > 0.05). (DOCX) [file pone.0304837.s004.docx]

**S3 Table: CCA descriptive statistics for Indonesian sites.** Canonical Correspondence Analysis (CCA) descriptive statistics for the interaction between all questionnaire responses in Indonesian sites. An asterisk (*) represents statistically significant factors (p > 0.05).

| Factor | CCA1 | CCA2 |
| --- | --- | --- |
| Socio-Demographics : Attitudes | | |
| Age | 0.162 | 0.217 |
| Household Size | 0.214 | 0.466 |
| Education Level | -0.071 | 0.185 |
| Percentage Income from Agriculture | 0.460 | -0.134 |
| Total Monthly Income* | -0.360 | -0.350 |
| Monthly Income Per HA | 0.264 | -0.195 |
| Years On Land | 0.171 | 0.050 |
| Village_BL | 0.155 | -0.011 |
| Village_BR | -0.678 | -0.056 |
| Village_GS | -0.145 | 0.481 |
| Village_KA | 0.293 | -0.154 |
| Village_KB | 0.038 | 0.277 |
| Village_KJ | -0.361 | -0.133 |
| Village_KM | 0.167 | -0.181 |
| Village_LJ | 0.041 | 0.150 |
| Village_PB | -0.083 | -0.130 |
| Village_PTP | -0.061 | 0.062 |
| Village_SB | 0.064 | -0.127 |
| Village_SL | -0.078 | 0.109 |
| Village_SM | 0.069 | 0.117 |
| Female | 0.147 | 0.060 |
| No involvement in other industry | 0.263 | 0.121 |
| Socio-demographics : Management Inputs* | | |
| Age | -0.012 | -0.117 |
| Household Size* | 0.437 | 0.120 |
| Education Level | -0.015 | -0.215 |
| Percentage Income from Agriculture | 0.006 | -0.267 |
| Total Monthly Income | 0.013 | -0.375 |
| Monthly Income from OP Per HA* | -0.507 | -0.006 |
| Years On Land | -0.149 | 0.245 |
| Village_BL | -0.131 | -0.068 |
| Village_BR | 0.039 | 0.063 |
| Village_GS | -0.168 | -0.154 |
| Village_KA | 0.003 | 0.166 |
| Village_KB | -0.105 | 0.054 |
| Village_KJ | -0.036 | 0.250 |
| Village_KM | -0.189 | -0.051 |
| Village_LJ* | 0.857 | -0.010 |
| Village_PB | 0.146 | -0.133 |
| Village_PTP | -0.143 | -0.193 |
| Village_SB | -0.261 | -0.059 |
| Village_SL | -0.030 | 0.403 |
| Village_SM | -0.169 | -0.222 |
| Female | 0.196 | -0.274 |
| No involvement in other industry | -0.082 | -0.384 |
| Attitudes : Management Inputs | | |
| Importance of Nature_Economic | -0.150 | 0.238 |
| Importance of Nature_Food | 0.177 | 0.088 |
| Importance Nature_Wildlife | -0.384 | -0.192 |
| Importance Nature_Beauty | 0.088 | -0.013 |
| Importance of Nature_Culture | 0.100 | -0.323 |
| Importance of Nature_Health | 0.309 | 0.080 |
| Influence on Management _Neighbours | 0.213 | 0.085 |
| Influence on Management _Scientific | -0.493 | 0.293 |
| Influence on Management _Cost | -0.137 | 0.035 |
| Influence on Management _Effort | -0.197 | -0.065 |
| Influence on Management _Consistency | -0.069 | -0.072 |
| Influence on Management _Yields | -0.030 | -0.048 |
| Preference for Agricultural Industry | -0.106 | -0.157 |
| Attitudes on Farming_Ease and sustainability | 0.118 | -0.022 |
| Herbicide Motivation_Weeds | 0.081 | -0.047 |
| Chemical Motivation_Pests | 0.246 | 0.173 |
| Fertilizer Type Motivation_Supplier | -0.302 | -0.095 |
| Favourite Animal_Cobra | -0.213 | -0.067 |
| Favourite Animal_Dragonflies and damselflies | 0.284 | 0.202 |
| Favourite Animal_Leopard cat | 0.041 | 0.063 |
| Favourite Animal_Yellow crazy ant | -0.137 | -0.084 |
| Least Favourite Animal_Bagworm caterpillar | -0.204 | -0.079 |
| Least Favourite Animal_Cobra | 0.276 | 0.400 |
| Least Favourite Animal_Long tailed macaque | 0.138 | -0.019 |
| Least Favourite Animal_Monitor lizard | -0.287 | 0.086 |
| Least Favourite Animal_N/A | -0.094 | 0.179 |
| Least Favourite Animal_Nettle caterpillar | -0.054 | 0.325 |
| Least Favourite Animal_Phyton | -0.061 | -0.011 |
| Least Favourite Animal_Rhinoceros beetle | 0.013 | -0.184 |
| Least Favourite Animal_Wild pig | 0.064 | -0.178 |
