# Supplementary material for: Private, non-profit, and plantation: Oil palm smallholders in management-assistance programs vary in socio-demographics, attitudes, and management practices
Source: PLoS One. 2025 Jan 17;20(1):e0304837. doi: 10.1371/journal.pone.0304837 (PMC11741574; doi:10.1371/journal.pone.0304837)
Supplement: S4 Table — Principal Component Analysis (PCA) descriptive statistics for Axes 1 and 2 of all questionnaire responses in Malaysian sites. (DOCX) [file pone.0304837.s005.docx]

**S4 Table: PCA descriptive statistics for Malaysian sites.** Principal Component Analysis (PCA) descriptive statistics for Axes 1 and 2 of all questionnaire responses in Malaysian sites.

| Factor | PC1 | PC2 |
| --- | --- | --- |
| Socio-demographics | | |
| Age | 0.086 | -0.027 |
| Household Size | -0.036 | -0.111 |
| Percentage Income From Agriculture | -0.149 | 0.078 |
| Total Monthly Income | 0.193 | -0.337 |
| Monthly Income From OP Per HA | 0.069 | -0.076 |
| Years On Land | 0.059 | 0.250 |
| District_BP | 0.155 | 0.324 |
| District_HP | 0.091 | -0.300 |
| District_K | 0.036 | 0.023 |
| District_PT | -0.188 | -0.220 |
| Married | -0.050 | -0.331 |
| Single | 0.050 | 0.331 |
| Female | -0.153 | -0.251 |
| Male | 0.153 | 0.251 |
| No Formal Education | -0.088 | 0.176 |
| Furthest Education_1 | -0.088 | -0.079 |
| FurthestEducation_2 | -0.164 | 0.011 |
| FurthestEducation_3 | 0.026 | 0.026 |
| FurthestEducation_4 | 0.015 | -0.075 |
| FurthestEducation_5 | 0.141 | 0.163 |
| FurthestEducation_6 | -0.136 | -0.048 |
| FurthestEducation_7 | 0.091 | -0.300 |
| OtherEmployment_Y | 0.248 | -0.130 |
| Not Land Owner | -0.384 | 0.029 |
| LandownerStatus_Y | 0.384 | -0.029 |
| Landowner Name_JAKOA | -0.380 | -0.005 |
| Landowner Name_Perak State Government | -0.043 | 0.106 |
| Landowner | 0.384 | -0.029 |
| Attitudes | | |
| Importance of Nature_Economic | -0.249 | 0.081 |
| Importance of Nature_Food | -0.152 | 0.140 |
| Importance of Nature_Wildlife | -0.184 | 0.042 |
| Importance of Nature_Beauty | -0.250 | 0.033 |
| Importance of Nature_Culture | -0.237 | 0.055 |
| Importance of Nature_Health | -0.208 | 0.144 |
| Importance Nature_None | 0.134 | 0.031 |
| Influence on Management _Neighbours | -0.139 | 0.090 |
| Influence on Management _Scientific | -0.223 | 0.095 |
| Influence on Management _Cost | -0.235 | 0.092 |
| Influence on Management _Effort | -0.161 | 0.085 |
| Influence on Management _Consistency | -0.082 | 0.242 |
| Influence on Management _Yields | -0.178 | 0.087 |
| Preference for Agricultural Industry | -0.112 | -0.084 |
| Herbicide Motivation_Weeds | 0.120 | 0.321 |
| Herbicide Motivation_Season | 0.230 | -0.031 |
| Herbicide Motivation_Money | 0.028 | -0.183 |
| Chemical Motivation_Pests | 0.012 | 0.214 |
| Chemicals Motivation_Other | 0.168 | 0.169 |
| Chemicals Motivation_Weeds | -0.093 | -0.275 |
| Motivation for Fertilizer Type_Supplier | 0.104 | 0.219 |
| Motivation for Fertilizer Type_Cooperative | -0.120 | 0.146 |
| Motivation for Fertilizer Type_Neighbors | -0.042 | -0.196 |
| FavoriteAnimal_Butterflies | -0.166 | -0.050 |
| Favourite Animal_Dragonflies and Damselflies | 0.072 | 0.002 |
| Favourite Animal_Feral dogs | 0.051 | 0.053 |
| Favourite Animal_Yellow crazy ant | 0.155 | 0.012 |
| Least Favourite Animal_Bagworm caterpillars | 0.024 | -0.030 |
| Least Favourite Animal_Cobra | 0.059 | 0.011 |
| Least Favourite Animal_Long tailed macaque | 0.137 | 0.022 |
| Least Favourite Animal_None | 0.040 | -0.020 |
| Least Favourite Animal_Rat | 0.118 | -0.089 |
| Least Favourite Animal_Rhinoceros | 0.092 | -0.064 |
| Least Favourite Animal_Wild pig | -0.221 | 0.086 |
| Reason for Favourite Animal_Beauty | -0.139 | -0.087 |
| Reason for Favourite Animal_Ecosystem | -0.046 | 0.094 |
| Reason for Favourite Animal_Yield | 0.210 | 0.012 |
| Reason for Least Favourite Animal_None | 0.040 | -0.020 |
| Reason for Least Favourite Animal_Yield | -0.040 | 0.020 |
| Management Inputs | | |
| Palms Per Hectare | -0.065 | 0.021 |
| Hours Farming Weekly Per HA | -0.006 | 0.151 |
| Number of Herbicide Types | -0.166 | -0.205 |
| Herbicide Applications Annual | -0.252 | 0.080 |
| Herbicide Cost Annual | -0.217 | -0.047 |
| Herbicide Cost Per HA Annual | -0.223 | 0.074 |
| Herbicide Litres Annual | -0.239 | -0.025 |
| Herbicide Litres Per HA Annual | -0.228 | 0.082 |
| ChemicalApplicationAnnual | -0.178 | 0.164 |
| Number of Fertilizer Types | -0.049 | -0.133 |
| Fertilizer Cost Per HA Annual | -0.105 | 0.234 |
| Fertilizer Amount Per HA Annual | -0.107 | 0.195 |
| Number of OP Harvests Monthly | -0.036 | 0.028 |
| No use of herbicide | 0.262 | 0.112 |
| Use of Herbicide | -0.262 | -0.112 |
| No Other Vegetation Control | -0.143 | 0.359 |
| Method Vegetation Clearing_Cut | -0.090 | -0.286 |
| Method Vegetation Clearing_Grass Machine | 0.213 | -0.079 |
| Method Vegetation Clearing_None | -0.143 | 0.359 |
| Herbicide Location_Circle | -0.233 | -0.131 |
| Herbicide Location_Path | -0.213 | -0.093 |
| Herbicide Location_Random | 0.018 | 0.143 |
| Herbicide Location_Multiple | 0.061 | -0.027 |
| No Use Of Fronds | 0.135 | 0.010 |
| Stacked Fronds | -0.135 | -0.010 |
| No Livestock Present | -0.129 | -0.135 |
| Livestocks Present | 0.129 | 0.135 |
| No Fertilizer Use | 0.112 | 0.025 |
| No Organic Manure Use | -0.105 | -0.051 |
| Organic Manure Use | 0.105 | 0.051 |
| No Intercropping | -0.166 | -0.028 |
| Intercropping | 0.166 | 0.028 |
| Animal Prevention Method_Barrier | -0.015 | -0.264 |
| Animal Prevention Method_Poison | 0.087 | -0.015 |
| Animal Prevention Method_Repel when seen | -0.023 | 0.259 |
