# Supplementary material for: Private, non-profit, and plantation: Oil palm smallholders in management-assistance programs vary in socio-demographics, attitudes, and management practices
Source: PLoS One. 2025 Jan 17;20(1):e0304837. doi: 10.1371/journal.pone.0304837 (PMC11741574; doi:10.1371/journal.pone.0304837)
Supplement: S5 Table — Full table of highest Canonical Correspondence Analysis (CCA) factor loadings for dependent factors in Malaysian sites. (DOCX) [file pone.0304837.s006.docx]

**S5 Table: CCA factor loadings for Malaysian sites.** Full table of highest Canonical Correspondence Analysis (CCA) factor loadings for dependent factors in Malaysian sites.

| Factor | CCA1 | CCA2 |
| --- | --- | --- |
| Attitudes affected by socio-demographics | | |
| ImportanceNature_Economic | -0.064 | 0.065 |
| ImportanceNature_Food | 0.066 | 0.057 |
| ImportanceNature_Wildlife | 0.045 | 0.041 |
| ImportanceNature_Beauty | -0.032 | 0.021 |
| ImportanceNature_Culture | -0.053 | 0.081 |
| ImportanceNature_Health | 0.036 | 0.031 |
| ImportanceNature_None | 0.147 | -0.075 |
| ManagementInfluence_Neighbours | -0.114 | -0.156 |
| ManagementInfluence_Scientific | -0.094 | 0.067 |
| ManagementInfluence_Cost | -0.102 | -0.050 |
| ManagementInfluence_Effort | -0.110 | -0.159 |
| ManagementInfluence_Consistancy | 0.066 | 0.029 |
| ManagementInfluence_Yields | 0.022 | -0.007 |
| AgriculturalPreference_N | 1.416 | 0.438 |
| AgriculturalPreference_Y | -0.150 | -0.097 |
| HerbicideMotivation_Weeds_N | -0.737 | -0.219 |
| HerbicideMotivation_Weeds_Y | 0.256 | 0.024 |
| HerbicideMotivation_Season_N | -0.327 | 0.125 |
| HerbicideMotivation_Season_Y | 1.638 | -0.590 |
| HerbMotivation_Money_N | 0.140 | 0.035 |
| HerbMotivation_Money_Y | -0.942 | -0.871 |
| HerbicideMotivation_Neighbors_N | 0.074 | -0.021 |
| HerbicideMotivation_Spontaneous_N | 0.074 | -0.021 |
| ChemicalMotivation_Pests_N | 0.033 | 0.224 |
| ChemicalMotivation_Pests_Y | 0.176 | -0.631 |
| ChemicalMotivation_Disease_N | 0.074 | -0.021 |
| ChemicalsMotivation_Other_N | -0.635 | -0.116 |
| ChemicalsMotivation_Other_Y | 0.329 | 0.014 |
| ChemicalsMotivation_Weeds_N | 0.130 | -0.010 |
| ChemicalsMotivation_Weeds_Y | -0.328 | -0.099 |
| FertilizerTypeMotivation_Supplier_N | -0.352 | -0.099 |
| FertilizerTypeMotivation_Supplier_Y | 0.244 | 0.011 |
| FertilizerTypeMotivation_Cooperative_N | 0.151 | 0.035 |
| FertilizerTypeMotivation_Cooperative_Y | -0.270 | -0.266 |
| FertilizerTypeMotivation_Neighbor_N | 0.081 | -0.019 |
| FertilizerTypeMotivation_Neighbor_Y | -0.302 | -0.089 |
| FavoriteAnimal_Butterflies | -0.278 | -0.240 |
| FavoriteAnimal_Dragonflies | 2.844 | -3.897 |
| FavoriteAnimal_Feral | 0.357 | 1.855 |
| FavoriteAnimal_Yellow | 2.300 | 0.219 |
| LeastFavoriteAnimal_Bagworm | 0.519 | -0.090 |
| LeastFavoriteAnimal_Cobra | 0.299 | -1.685 |
| LeastFavoriteAnimal_Long | 2.307 | -1.160 |
| LeastFavoriteAnimal_None | 0.519 | 1.206 |
| LeastFavoriteAnimal_Rat | 0.177 | -1.688 |
| LeastFavoriteAnimal_Rhinoceros | 1.689 | 0.354 |
| LeastFavoriteAnimal_Wild | -0.392 | 0.155 |
| ReasonFavoriteAnimal_Beauty | -0.290 | -0.475 |
| ReasonFavoriteAnimal_Ecosystem | -0.026 | 1.060 |
| ReasonFavoriteAnimal_Yield | 1.089 | 0.152 |
| ReasonLeastFavoriteAnimal_None | 0.519 | 1.206 |
| ReasonLeastFavoriteAnimal_Yield | 0.064 | -0.046 |
| Management inputs affected by socio-demographics | | |
| PalmsPerHectare | -0.338 | -0.220 |
| HoursFarmingWeeklyPerHA | -0.371 | 0.260 |
| NoHerbicideTypes | -0.483 | -0.287 |
| HerbicideApplicationsAnnual | -0.286 | -0.038 |
| HerbicideCostAnnual | -0.527 | -0.135 |
| HerbicideCostPerHAAnnual | -0.757 | 0.315 |
| HerbicideLitersAnnual | -0.517 | -0.077 |
| HerbicideLitersPerHAAnnual | -0.728 | 0.359 |
| ChemicalApplicationAnnual | -0.247 | 0.005 |
| NoFertilizerTypes | -0.283 | -0.270 |
| FertilizerCostPerHAAnnual | 0.169 | 0.085 |
| FertilizerAmountPerHAAnnual | 0.057 | -0.205 |
| NoOPHarvestsMonthly | -0.293 | -0.165 |
| UseOfHerbicide_N | 0.025 | 0.367 |
| UseOfHerbicide_Y | -0.380 | -0.257 |
| OtherVegetationControl_N | -0.100 | 0.228 |
| OtherVegetationControl_Y | -0.420 | -0.342 |
| MethodVegetationClearing_Cut. | -1.084 | -0.523 |
| MethodVegetationClearing_Grass.Machine | -0.009 | -0.230 |
| MethodVegetationClearing_None | -0.100 | 0.229 |
| HerbicideLocation_Circle_N | 0.076 | -0.312 |
| HerbicideLocation_Circle_Y | -0.553 | -0.084 |
| HerbicideLocation_Path_N | -0.037 | -0.088 |
| HerbicideLocation_Path_Y | -0.488 | -0.214 |
| HerbicideLocation_Random_N | -0.406 | -0.197 |
| HerbicideLocation_Random_Y | 0.277 | 0.039 |
| HerbicideLocation_All_N | -0.339 | -0.044 |
| HerbicideLocation_All_Y | -0.057 | -2.069 |
| UseOfFronds_None | 0.229 | -0.199 |
| UseOfFronds_Stacked | -0.345 | -0.166 |
| LivestockPresence_N | -0.422 | -0.244 |
| LivestockPresence_Y | 0.276 | 0.291 |
| FertilizerUse_N | 0.154 | 2.529 |
| FertilizerUse_Y | -0.332 | -0.224 |
| OrganicManureUse_N | -0.353 | -0.534 |
| OrganicManureUse_Y | -0.281 | 0.321 |
| Intercropping_N | -0.332 | -0.224 |
| Intercropping_Y | -0.211 | 0.466 |
| AnimalPreventionMethod_Barrier | -0.955 | -0.734 |
| AnimalPreventionMethod_Poison | 0.389 | 0.003 |
| AnimalPreventionMethod_Repel | -0.122 | 0.039 |
| Management inputs affected by attitudes | | |
|  | CCA1 | CCA2 |
| PalmsPerHectare | -0.252 | -0.546 |
| HoursFarmingWeeklyPerHA | -0.219 | -0.576 |
| NoHerbicideTypes | -0.452 | -0.184 |
| HerbicideApplicationsAnnual | -0.321 | 0.009 |
| HerbicideCostAnnual | -0.687 | 0.035 |
| HerbicideCostPerHAAnnual | -0.797 | 0.156 |
| HerbicideLitersAnnual | -0.637 | 0.084 |
| HerbicideLitersPerHAAnnual | -0.747 | 0.198 |
| ChemicalApplicationAnnual | -0.234 | -0.293 |
| NoFertilizerTypes | -0.213 | -0.534 |
| FertilizerCostPerHAAnnual | 0.169 | 0.054 |
| FertilizerAmountPerHAAnnual | 0.109 | -0.068 |
| NoOPHarvestsMonthly | -0.220 | -0.537 |
| UseOfHerbicide_N | 0.759 | -3.666 |
| UseOfHerbicide_Y | -0.373 | -0.087 |
| OtherVegetationControl_N | -0.269 | 0.087 |
| OtherVegetationControl_Y | -0.186 | -0.901 |
| MethodVegetationClearing_Cut. | -0.936 | -0.597 |
| MethodVegetationClearing_Grass.Machine | 0.279 | -1.089 |
| MethodVegetationClearing_None | -0.269 | 0.087 |
| HerbicideLocation_Circle_N | 0.210 | -1.477 |
| HerbicideLocation_Circle_Y | -0.456 | -0.089 |
| HerbicideLocation_Path_N | 0.222 | -1.491 |
| HerbicideLocation_Path_Y | -0.463 | -0.080 |
| HerbicideLocation_Random_N | -0.249 | -0.698 |
| HerbicideLocation_Random_Y | 0.058 | 0.112 |
| HerbicideLocation_All_N | -0.196 | -0.619 |
| HerbicideLocation_All_Y | -0.438 | -0.282 |
| UseOfFronds_None | 1.392 | -4.274 |
| UseOfFronds_Stacked | -0.279 | -0.442 |
| LivestockPresence_N | -0.329 | -0.513 |
| LivestockPresence_Y | 0.497 | -1.113 |
| FertilizerUse_N | -0.650 | -4.258 |
| FertilizerUse_Y | -0.202 | -0.522 |
| OrganicManureUse_N | -0.171 | -0.469 |
| OrganicManureUse_Y | -0.264 | -0.771 |
| Intercropping_N | -0.222 | -0.438 |
| Intercropping_Y | -0.084 | -2.401 |
| AnimalPreventionMethod_Barrier | -0.754 | -0.711 |
| AnimalPreventionMethod_Poison | 0.725 | -1.914 |
| AnimalPreventionMethod_Repel | -0.058 | -0.478 |
