# Supplementary material for: Private, non-profit, and plantation: Oil palm smallholders in management-assistance programs vary in socio-demographics, attitudes, and management practices
Source: PLoS One. 2025 Jan 17;20(1):e0304837. doi: 10.1371/journal.pone.0304837 (PMC11741574; doi:10.1371/journal.pone.0304837)
Supplement: S6 Table — Canonical Correspondence Analysis (CCA) descriptive statistics for the interaction between all questionnaire responses in Malaysian sites. An asterisk (*) represents statistically significant factors (p > 0.05). (DOCX) [file pone.0304837.s007.docx]

**S6 Table: CCA descriptive statistics for Malaysian sites.** Canonical Correspondence Analysis (CCA) descriptive statistics for the interaction between all questionnaire responses in Malaysian sites. An asterisk (*) represents statistically significant factors (p > 0.05).

| Factor | CCA1 | CCA2 |
| --- | --- | --- |
| Socio-demographics : Attitudes* | | |
| Age | 0.204 | 0.564 |
| Household Size | 0.093 | 0.024 |
| Percentage Income From Agriculture | 0.454 | 0.163 |
| Total Monthly Income | 0.212 | 0.010 |
| Monthly Income From OP Per HA | 0.182 | 0.198 |
| Years On Land | -0.133 | 0.265 |
| District_BP* | -0.480 | 0.122 |
| District_HP | 0.118 | 0.032 |
| District_K* | -0.434 | 0.233 |
| Married | 0.398 | -0.136 |
| Female | 0.225 | 0.069 |
| No Formal Education | 0.053 | 0.242 |
| Furthest Education_1 | 0.286 | 0.141 |
| Furthest Education_2* | -0.039 | -0.477 |
| Furthest Education_3 | -0.250 | -0.161 |
| Furthest Education_6 | -0.027 | -0.334 |
| No Other Employment | 0.147 | 0.146 |
| Not Landowner | 0.178 | -0.481 |
| Landowner Name_Perak State Government | 0.007 | -0.131 |
| Socio-demographics : Management inputs | | |
| Age | 0.325 | 0.133 |
| Household Size | 0.058 | -0.067 |
| Percentage Income From Agriculture | 0.010 | -0.048 |
| Total Monthly Income | 0.040 | -0.155 |
| Monthly Income From OP Per HA | 0.182 | 0.434 |
| Years On Land | -0.043 | -0.056 |
| District_BP | -0.269 | 0.112 |
| District_HP | -0.101 | -0.587 |
| District_K | 0.372 | -0.121 |
| Married | 0.174 | -0.373 |
| Female | -0.090 | -0.272 |
| No Formal Education* | -0.341 | 0.170 |
| Furthest Education_1 | -0.131 | 0.083 |
| Furthest Education_2 | -0.193 | -0.320 |
| Furthest Education_3 | 0.100 | -0.175 |
| Furthest Education_6 | -0.136 | -0.225 |
| No Other Employment | -0.085 | -0.143 |
| Not Landowner | -0.383 | -0.269 |
| Landowner Name_Perak State Government | -0.676 | 0.196 |
| Attitudes : Management inputs* | | |
| Importance of Nature_Economic | -0.191 | -0.313 |
| Importance of Nature_Wildlife | -0.209 | -0.188 |
| Importance of Nature_Beauty | -0.304 | -0.281 |
| Importance of Nature_Culture | -0.282 | -0.393 |
| Importance of Nature_None | -0.090 | 0.322 |
| Influence on Management _Neighbours | 0.000 | -0.115 |
| Influence on Management _Cost | -0.100 | -0.203 |
| Influence on Management _Effort | 0.054 | -0.125 |
| Influence on Management _Consistancy | 0.058 | -0.091 |
| Influence on Management _Yields | 0.186 | -0.012 |
| Preference for Agricultural Industry | -0.029 | -0.194 |
| Herbicide Motivation_Weeds | -0.201 | 0.516 |
| Herbicide Motivation_Season | 0.207 | 0.412 |
| Herbicide Motivation_Money | -0.207 | -0.174 |
| Chemical Motivation_Pests | 0.174 | 0.299 |
| Motivation for Fertilizer Type_Cooperative | -0.003 | 0.055 |
| Motivation for Fertilizer Type_Neighbors | 0.246 | 0.085 |
| FavoriteAnimal_Butterflies | -0.125 | -0.108 |
| Favourite Animal_Dragonflies and Damselflies | 0.315 | 0.178 |
| Favourite Animal_Feral dogs | -0.126 | -0.302 |
| Least Favourite Animal_Bagworm caterpillars | -0.351 | 0.082 |
| Least Favourite Animal_Long tailed macaque | 0.269 | 0.229 |
| Least Favourite Animal_None | -0.123 | 0.004 |
